# Extraction of keratin particles as intact protein sequences from chicken feathers and their characterization

**DOI:** 10.1016/j.bbiosy.2026.100128

**Published:** 2026-01-17

**Authors:** Julia Chuttke, Luisa Scholz, Johannes Wohlrab, Mandy Koch, Gerd Hause, Matthew Fuszard, Adina Eichner

**Affiliations:** a Department of Dermatology and Venereology, Martin Luther University Halle-Wittenberg, Ernst-Grube-Str. 40, 06120 Halle, Germany; b Institute of Applied Dermatopharmacy at Martin Luther University Halle-Wittenberg, Weinbergweg 23, 06120 Halle, Germany; c Institute of Chemistry, Food Chemistry, Martin Luther University Halle-Wittenberg, Kurt-Mothes-Str. 2, 06120 Halle, Germany; d Microscopy Unit, Biocenter, Martin Luther University Halle-Wittenberg, Weinbergweg 22, 06120 Halle, Germany; e Interfaculty Core Facility – Proteomic Mass Spectrometry, Charles Tanford Protein Center, Martin Luther University Halle-Wittenberg, Kurt-Mothes-Str. 3a, Halle, Germany

**Keywords:** Feather keratin, Protein extraction, Protein characterization, Cell culture assays, HET-CAM

## Abstract

Keratins are ubiquitously occurring proteins, which are the structural basis of e.g. hairs, nails, and even skin. In detail, the Stratum corneum (SC) barrier function depends on the keratin-filled corneocytes and the surrounding lipid matrix. Our focus was on keratins as artificial substituents and model substances for/on damaged or irritated SC with limited barrier properties. We were able to extract full-sequenced, intact fibrous proteins from chicken feathers using a urea and L-cysteine-based extraction method, with the intention to avoid the application of irritating sodium dodecyl sulfate and final keratin precipitation. Keratin particles with a high degree of water-solubility were received. Typical characteristics of feather keratins were further verified: FT-IR technique revealed the presence of α-helical structures and β-sheets. Applying gel electrophoresis techniques, a main fraction was observed with a molecular mass of 10 kDa. Finally, mass spectrometry identified feather keratins with 10.1 kDa and 98 amino acids, indicating the complete protein sequences. In aqueous dispersion, a DLS study revealed that keratin particles were in a colloidal state with an average particle size of about 300 nm and a zeta potential of –40 mV. Cell proliferation and cell vitality tests on juvenile native human dermal fibroblasts and native human epidermal keratinocytes cells presented the physiological effect of the keratin particles. A final *ex vivo* study on hen´s eggs revealed no irritative potential of the keratin, which was crucial for the intended usage on damaged or irritated SC.

## Highlights

• Keratin particles extracted from chicken feathers were successfully produced.
• Mass spectrometry identified four feather keratins with a molecular mass of 10 kDa.
• A filament structure of the proteins was observed by microscopic technique TEM.
• No toxic effect was observed for keratins in cell proliferation and vitality tests.
• HET-CAM test showed no irritative potential of keratin particles derived.

## Graphical abstract

**Figure:**
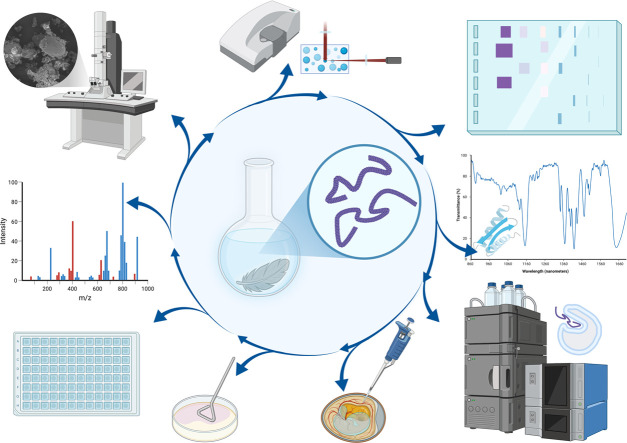

## 1 Introduction

Keratins and keratin-associated proteins are a heterogeneous group of cysteine-rich proteins, featuring a high degree of sulfur [1,2]. Thereby, the high numbers of disulfide bonds are stabilizing keratin-rich natural structures as wool, feathers, nails, hairs, horns, hooves, hedgehog spines, claws, or whale baleen. Mostly, keratins were derived from sheep wool. Overall, feathers became a worthwhile source of keratins in the last years, as over 65 million tons are available as waste product from the poultry industry [[3], [4], [5], [6]]. As fibrous polymers, keratins were introduced as multifunctional, renewable biomaterials, particularly due to their high degree of resilience, compatibility, and biological degradability [[7], [8], [9]]. In detail, keratins and numerous keratin-related modifications were improved as films or coatings, fibers, hydrogels, and foams [7,[10], [11], [12], [13], [14]]. Depending on their amount of sulfur, they are described as hard or soft keratins [15]. Filaments of hard keratin are well-ordered and highly stable based on additional disulfide bonds featuring a high number of crosslinks. A limited degree of sulfur results in so-called soft keratin fibers, loosely structured filaments in the cytoplasm. Moreover, the secondary structure can be consulted to characterize the several keratins. According to that, α -helical dominated keratins are named as alpha-keratins, whereby a secondary structure of β-sheets leads to a description as beta-keratins. Alpha-keratins can be derived from hairs, wool, horn-based materials as nails or the Stratum corneum as the outermost layer of human skin. Besides, beta-keratin sources are feathers, bird claws and reptile shells [16]. As a third kind of keratins, feather keratins can be interpreted, where both configurations of the polypeptide chains are available, α-helices and β-sheets [[17], [18], [19]]. Here, non-flexible and non-elastic feather keratins led to stiff but light feathers [18,20]. However, with about 7-8%, feather keratins have a smaller cysteine ratio than human hair (17-18%) [[21], [22], [23], [24]]. As numerous natural keratin sources are known, the extraction procedures differ depending on the raw material and the kind of extracted keratins. In detail, common extraction procedures use oxidation [25], bases [26] and/or sodium-based ionic surfactants [27,28] or microwaves [29]. Moreover, there are keratin degradation methods established, which are based on enzymes or microorganisms [[30], [31], [32], [33], [34]]. Extraction procedures based on chemical reduction applied sulfur-containing agents as 2-mercaptoethanol and other thiols alone or in combination with sodium dodecyl sulfate (SDS) [[35], [36], [37], [38], [39]]. Urea was described as excipient to increase the degree of keratins´ water solubility [40]. Keratin extraction due to the so-called “Shindai method” facilitated yields of more than 65 % [27,[41], [42], [43]], mostly derived from hair. However, the extraction of the intact protein is difficult due to high temperatures and/or denaturing pH conditions [[44], [45], [46]] resulting in feather keratin lysates [47]. Based on a method described by *Martelli et al.* [48], β-keratin from chicken feathers was extracted using a combination of reducing agent 2-mercaptoethanol together with urea and SDS. The extraction of keratins from chicken feathers was described by *Fujii and Li* before [43], who developed a method for the extraction of the protein from hair, nail, and wool based on the “Shindai method” in order to produce protein films. They used a combination of Tris-HCL (pH 8.5), thiourea, urea and 2-mercaptoethanol for the extraction. Another work presented a combination of urea, SDS, sodium metabisulphite and natrium hydroxide at pH 6.5, where the extracted keratin dispersion was used for the production of nanofibers by electrospinning [49]. The group of *Yiqi Yang* proposed several keratin extraction procedures from chicken feathers using a L-cysteine and SDS-based method, with keratin potentially be applied as new fiber for textiles or further material sciences [50,51]. In recent works, they avoided SDS, whereby the keratins extracted from duck feathers nevertheless precipitated, which led to bio-inactive keratins [52,53]. However, since no keratins are commercially available as full sequenced proteins and most of the extraction methods included toxic or irritating chemicals that are difficult to remove afterwards and ultimately resulted in hydrolyzed peptides from precipitates, an environmentally friendly and gentle extraction process is needed, in the best case avoiding the current problem of raw material shortages in various supply chains at the same time. In the present work a gentle extraction of keratins from chicken feathers avoiding any carcinogenic, mutagenic or irritating agents was aimed with respect to a non-irritating powder for e.g. application on or as a model for damaged or sensible skin. Especially, the structure of the primary and secondary structure of the proteins should be maintained and the hydrolyzation into keratin peptides should be prevented. For this purpose, a complex procedure was tested, where L-cysteine was reducing the disulfide bond networks for higher yield. Afterwards, the product was identified by mass spectrometry (MS) and Fourier transform infrared spectroscopy (FT-IR) and characterized comprehensively: 2D SDS-PAGE (two-dimensional SDS polyacrylamide gel electrophoresis) was used to determine the molecular sizes and the isoelectric points. Microscopic techniques were applied to reveal the particle size of the powder via scanning electron microscope (SEM) and of the keratin particles in colloidal dispersion via transmission electron microscopy (TEM). Besides, dynamic light scattering (DLS) studies should present the particle size of keratin in colloidal dispersion as well. Size exclusion chromatography (SEC) coupled with multi-angle light scattering (MALLS) experiments were performed with the aim to determine the molecular weight of the colloidal keratins in dispersion. The investigation of the zeta potential should characterize the agglomeration behavior of the keratins´ dispersion, which will be surveyed as a function of the pH. The physiological and absence of toxic effects of the keratin particles were investigated in cell culture assays on primary fibroblasts and keratinocytes. Here, normal human epidermal keratinocytes (NHEK) and normal human dermal fibroblasts (NHDF) were used for this purpose. A HET-CAM study should reveal the effect of the keratins on *ex vivo* eukaryotic cells located in the chorioallantoic membranes (CAM) of hen’s eggs (HET). Together with a final microbiological test following the European Pharmacopoeia (Ph. Eur.), aspects regarding the safety, purity and non-irritative potential of the keratins were aimed to be determined. Overall, the intention was to test the possibility to extract keratins as bio-intact full-sequenced proteins from chicken feather waste and to receive a safe and non-irritating keratin powder product. The selected powder form should ensure handling benefits with respect to pharmaceutical technology, stability, storage capacities and dosages. Especially in this case, its application as a sustainable substance used as, depending on its properties, humectant in cosmetic products or as pharmaceutical excipient on irritated skin or as a model substance for a keratolytic skin status as e.g., scabies in *in vitro* diffusion studies of pharmaceutical active ingredients (APIs) would be of high benefit with respect to the triangle of sustainability, costs for excipients and a worthful model substance.

## 2 Material and methods

### 2.1 Production of keratin particles

Chicken feathers were cleaned with soap and water, disinfected with 70% (v/v) ethanol (VWR CHEMICALS, Germany) and subsequently dried at room temperature. Afterwards the complete feathers were cut into small pieces (< 1 mm) and homogenized in a cutting mill (SM comfort, Retsch GmbH, Germany). The feather homogenate was transferred into a cellulose extraction thimble and degreased in a Soxhlet apparatus with petroleum ether (Carl Roth GmbH & Co. KG, Germany) at 65°C for 12 h. For the extraction of keratins, 7 g of the degreased feather material were added to 300 mL of an extraction buffer composed of: 10 M urea, 200 mM L-cysteine hydrochloride and 25 mM Tris-HCl (pH 8.5) (all chemicals Carl Roth GmbH & Co. KG, Germany) with a pH value of 10.5, and extracted at 65°C for 48 h. The extract was centrifuged at 3260 x g for 25 min and the supernatant filtrated two times. Then, the filtrate was transferred into dialysis membranes (Spectra/Por 3, Standard RC Tubing, MWCO: 3.5 kDa, Repligen) and dialyzed against DI water in exchange 1:200 to remove urea residues. The dialysate was processed into a powder by spray drying. Therefore, a lab spay dryer (B-290, Büchi GmbH, Germany) was used with the following parameter: inlet temperature 120°C, outlet temperature 100°C, condensation temperature 12°C, pump volume flow 0.4 L/h, gas volume flow 35 L/min. Finally, keratin particles in powder form were derived. Either the spray-dried keratin particles were directly applied in the following characterization studies or as colloidal dispersion in defined concentrations in DI water (see following sections for details).

### 2.2 Protein biochemical studies

#### 2.2.1 IEF/ 2D-SDS-PAGE

First isoelectric focusing (IEF) was performed prior to the sodium dodecyl sulfate − polyacrylamide gel electrophoresis (SDS−PAGE) in order to distinguish between different keratin fractions by their masses and isoelectric points (pI). The keratin concentration was 5.241 μg/μL and 19.1 μL of the sample dispersion were applied to the IGP strips (Immobiline Dry Strip 18 cm pH 3 – 10 NL, Cytiva Europe GmbH, Germany). The strips were covered with SERVA HPE™ IPG Overlay (Serva Electrophoresis GmbH, Germany) and incubated at 20°C for 12 h. An IEF100 Focusing Unit (Hoefer Inc., USA) was used for the electric focusing applying the following phases: phase 1 (step and hold, 500 V, 60 min), phase 2 (gradient, 1,000 V, 60 min) phase 3 (gradient, 10,000 V, 180 min) phase 4 (step and hold, 10,000 V, 18500 Vhr). For the second dimension, a 2D gel (dimensions 255 × 200 × 0.65 mm; 10 – 15% acrylamide gradient gel with NF backing) was used. For the equilibration, each 6 mL of two equilibration solutions E-DTT and E-IAA (E-DTT: 6 M urea, 50 mg 1,4 dithiothreit, 5 mL buffer (30% (v/v) glycerol, 50 mM Tris HCL (pH 8.8) (all chemicals were purchased from Carl Roth GmbH & Co. KG, Germany), 2% (w/v) SDS, 0.1 mM EDTA); E-IAA: 6 M urea, 125 mg IAA, 5 mL buffer (see buffer E-DTT)) were applied to the 2D gel and incubated for 15 min at 20°C prior to the sample loading. After sample and marker (PageRuler™ Plus Prestained Protein Ladder, ThermoFisher) loading, the SDS PAGE ORCA Gel Electrophoresis Modul (NH DyeAGNOSTICS GmbH, Germany) was programmed to the following steps: step 1 (100 V, 7 mA, 1 W, 30 min), step 2 (200 V, 13 mA, 3 W, 30 min), step 3 (300 V, 20 mA, 5 W, 10 min), step 4 (pause with removal von IPG strip from 2D gel), step 5 (220 V, 5 mA, 2 W, 14 hours), and step 6 (1,000 V, 40 mA, 30 W, 3 h). According to the protocol of Sigma-Aldrich [54], the staining procedure was performed using SYPRO® Ruby Protein Gel Stain. For the final imaging, a FLA9000 Fluorescence Scanner (Fuji® Film) with a high resolution of 100 µm was used and the detection was performed by a SYPRO® Ruby Laser LD473 with a LPG filter, a PMT of 700 V and a grey min to max of 26764 – 60928. The detection and export of the spots was possible using Delta2D-Software V4.8 (Decodon). To analyze the identity of the spots separated, selected spots were picked and subjected to gel digestion for further MS analysis (see Section 2.3.2 mass spectrometry).

### 2.3 Biophysical studies

#### 2.3.1 Fourier transform infrared spectroscopy (FT-IR spectroscopy)

The IR spectrum of the keratin powder was ascertained by using a Bruker spectrometer (Vertex 70, Bruker Optics, Germany) equipped with an attenuated total reflection (ATR) measuring module (Thermo Spectra-Tech, HATR attachment, USA). Therefore, five times 10 mg of the powder were pressed in a potassium bromide pellet. The samples were measured at 20°C by infrared light, which passes through a ZnSe crystal with a 20 mm diameter and a 45° incidence angle. Per sample, 32 scans were carried out.

#### 2.3.2 Mass spectrometry

For the protein digestion, the keratins were reduced and alkylated as previously described in standard MS techniques [55], however in order to maximize coverage of a protein that suffers from either a glut or scarcity of conventional protease cleavage sites (i.e., Lysine and Arginine for Trypsin and Aspartic acid and Glutamic acid for GluC), a process involving inefficient digestion was attempted thereby creating partially cleaved protein and significantly higher numbers of missed cleavage peptides. Samples were digested in suboptimal conditions (20°C) for a very short period (10 min) with Proteinase K before the reaction was inhibited immediately by adding 1% Trichloroacetic acid (TCA). As the mixture then contained undigested proteins, peptides were enriched with C18 pipette tips. The Nano-LC separation was performed as followed: For 1 h gradient analytical runs 100 ng of digested proteins were injected into the chromatography system. Peptides were separated on a Dionex UltiMate 3000 RSLCnano system consist of NCS-3500RS Nano ProFlow and WPS-3000TPL RS UPLC System (ThermoFisher, Germany) equipped with a PepMap™ Neo 5 µm C18 300 µm × 5 mm Trap cassette (ThermoFisher, Germany) as well as a ACQUITY UPLC M-Class Peptide BEH C18 Column, 130 Å, 1.7 µm, 75 µm × 250 mm (Waters, Germany). After injection of 50 μL, peptides were trapped for 10 min at 15 μL/min at 3% B (A: 0.1% formic acid in water, B: 0.1% formic acid in ACN) and separated at 240 nL/min in a linear gradient of 3-50% B within 50 min. For the mass spectrometric analysis, the eluting peptides were ionized at 2.1 kV and 320°C in a Exploris 480 ThermoFisher nanoESI source using a Pre-cut PicoTip Emitter; 360 μm OD × 20 μm ID, 10 μm tip; 12 cm long (CoAnn Technologies, LLC). Exploris 480 was running FAIMS with -50 CV, MS range of 350-1500 m/z, 1 second cycle time, 120000 Orbitrap resolution, with auto AGC settings. For the data analysis the .raw files were analyzed using the Byonic (v5.1.1 – Protein Metrics Inc, USA) search engine with the following settings: Cleavage site(s) was left blank to allow for a C-terminal cleavage at any site. Precursor mass tolerance was set at 10 ppm, and fragment tolerance set at 20 ppm. Both oxidation and carbamidomethylation were set to variable to allow for incomplete DTT reduction of the keratin disulfide bonds, and a ‘Wildcard’ search was enabled to check for any modification to a cysteine between the masses of -40 (mass loss) to +300 to allow for surviving S-S crosslinks with unknown flanking residues to the crosslinked cysteine. In addition, the Disulfide and Trisulfide xlink algorithms were activated in Byonic and set for Protein #1 in the *Gallus gallus* FASTA database file (accessed 20-10-2023; Keratin protein P20307 was moved to the first position in the database). Automatic peptide score cuts were activated, alongside a Protein FDR of 1%. Peptide matches were accepted with a *p* value < 0.01 and were additionally manually curated. With the aim, to calculate proportions of the keratin identified semi-quantitatively, a modified iBAQ principle was applied, whereby standard iBAQ scores were generated using the proportion (total peptide intensity)/(number of observable tryptic peptides) [56].

#### 2.3.3 Dynamic light scattering (DLS)

The size of the colloidal keratin particles was analyzed by dynamic light scattering (DLS) from *n* = 10 keratin batches. For the investigation, 1 mL of a 0.1 mM colloidal keratin dispersion (diluted in DI water), was transferred into a polystyrene single-use cuvette and subsequently applied into the tempered cuvette module of the Zetasizer (ZEN3600, Malvern Instruments, UK) at 25°C. The particle size was determined by use of the automatic analysis mode (general purpose) and back angle scattering at 173°. Each measurement was carried out as triplicate (*n* = 3) and always enfolded *n*= 15 measuring cycles per run. Afterwards the data per sample of the particle size and polydispersity index (PDI) were averaged arithmetically.

#### 2.3.4 Zeta potential

An electrophoretic light scattering was carried out to determine the charge of the dispersed keratin particles of *n*= 10 keratin batches per solvent. For this purpose, 1 mL of a 0.3 mM (diluted in 10 mM NaCl) and 1 mL of a 0.1 mM (diluted in DI water) colloidal keratin dispersion, were transferred into a single-use capillary cell, and applied into the cuvette module of the Zetasizer (ZEN3600, Malvern Instruments, UK) at 25°C. Keratin particles were measured as triplicates per batch (*n*= 3), whereas the number of measuring cycles was determined automatically by the software. Subsequently, the ascertained measuring data per sample were averaged arithmetically.

#### 2.3.5 pH titration

The isoelectric point of the colloidal keratin dispersion was determined by pH titration. Here, 1 mL of a 0.1 mM keratin dispersion (diluted in DI water) was analyzed in a single-use capillary cell with the Zetasizer Ultra (ZSU3305, Malvern Panalytical Limited, UK) at 25°C. The titration was realized in a pH range from 3 to 10 with a 0.5 pH sequence increment, which was controlled by the Zetasizer Ultra Software. To adjust the isoelectric point, the pH value was increased (pH 3-10) with a 0.25 M NaOH solution and decreased (pH 10-3) with a 0.025 M and 0.25 M HCl solution. The volumes of each base and acid needed for the pH adjustment were added automatically. The procedure was carried out as triplicate (*n*= 3) and to each measuring point the zeta potential and the particle size were ascertained. Finally, the determined measuring data per sample were averaged arithmetically. Additionally, samples of the titration solution in the pH range between 3 and 4 were collected and the particle sizes were recorded microscopically and determined by Photoshop CS5 (Adobe, Ireland). Due to the strong accumulation behavior of the keratins, the large particle size could not be detected with the Zetasizer Ultra. For the calculation of the sizes (*n*= 30 particles) per pH point (pH 3.5 and pH 3), the measuring data were averaged arithmetically.

#### 2.3.6 SEC-MALLS

SEC liquid chromatography separation was performed on an Agilent 1100 High Performance Liquid Chromatography (HPLC) system (Agilent, USA) equipped with an integrated degassing unit, binary pump, column compartment, autosampler and UV/VIS detector in order to gain additional information about the keratin particle sizes and their distribution. A TSKgel 5000PW_XL_ column (7.8 mm ID × 30 cm, 10 μm particle size, Tosoh Bioscience, Japan) or a TSKgel 3000PW_XL_ (7.8 mm ID × 30 cm, 10 μm particle size, Tosoh Bioscience, Japan) with preconnected guard column TSKguardcolumn PW_XL_ (6 mm ID x 4.0 cm, Tosoh Bioscience, Japan) were used for separation. The mobile phase was ultrapure water (containing 0.05% sodium azide) with a constant flow rate of 0.3 mL/min (for TSKgel 5000PW_XL_ column), 0.4 mL/min (for TSKgel 2500PW_XL_ column), and 0.45 mL/min (TSKgel 3000PW_XL_ column) at a column temperature of 40°C. With a constant flow rate of 0.4 mL/min, two TSKgel 2500PW_XL_ columns were coupled as well with the aim of a higher separation and peak resolution. A multi-angle laser light scattering detector (SLD 7100; PSS GmbH, Germany, angle 90°) and a refractive index (RI) detector (Agilent, USA) were used to determine the concentration and molecular size. The RI detector was tempered to 35°C. Three colloidal keratin samples (*n*= 3) in DI water were measured each in a concentration of 1 mg/mL.

### 2.4 Microscopic studies

#### 2.4.1 Scanning electron microscopy (SEM)

The scanning electron microscopy (SEM) was used to ascertain the particle sizes and microstructure of the keratin particles produced by spray drying. For the imaging procedure, 5 mg of the powder were coated with an ultra-thin palladium layer by evaporation, causing some particles to collapse. Afterwards the sample was analyzed in the SEM (Quanta 3D FEG from FEI, USA) equipped with an Everhart-Thornley detector. The images of the keratin powder were acquired at 5 kV and in different magnifications. To determine the size of the keratin powder, *n*= 100 particles from Fig. 2 B were measured by Photoshop CS5 (Adobe, Ireland).

#### 2.4.2 Transmission electron microscopy (TEM)

Transmission electron microscopy (TEM) was used to characterize the particle size and shape of the keratin particles in colloidal dispersion. Therefore, 2 µL of a 0.5 mg/mL colloidal keratin dispersion, diluted in DI water, were applied on a copper TEM grid covered with formvar. After three washing steps with DI water, the grids were placed in a uranyl acetate solution (1% in water; Chemapol, Czech Republic) to contrast the sample. Afterwards, the samples were analyzed with a Transmission electron microscope (EM 900, Carl Zeiss Microscopy, Germany), operating at 80 kV. The images were taken with a Variospeed SSCCD camera SM-1k-120 (TRS, Germany). The determination of the particle sizes was realized based on *n*= 25 colloidal particles in Fig. 2 D with Photoshop CS5 (Adobe, Ireland).

### 2.5 Biological studies

#### 2.5.1 Cell proliferation assay

The effects of keratin particles on a cellular level were analyzed based on the proliferation rate of relevant cell types after 24 h and 48 h treatment with keratin. Here, a cell proliferation ELISA (enzyme-linked immunosorbent assay) with bromodesoxyuridine (BrdU) (Roche Diagnostics GmbH, Germany) was used, whereby the BrdU incorporation during the DNA synthesis was ascertained by cell proliferation. The assays were realized on native human dermal fibroblasts (NHDF) and native human epidermal keratinocytes (NHEK) derived from juvenile foreskins of three donors. In each six wells of a 96-well plate, 100 µL of each cell suspension, with a concentration of 3 × 10^3^ cells/mL, were applied. Afterwards, the NHDF cells were incubated for 72 h and the NHEK cells for 96 h at 37°C and 5% CO_2_. The physiological impact of keratins was tested for both solid keratin particles and their colloidal dispersion in concentrations of 0.1%, 0.005%, 0.01%, 0.005% and 0.001%, for all in individual triplicates (*n* = 3). To perform the assays, the keratin treatment was stopped through elimination of the dispersion and addition of 100 µL of fresh cell medium. The cell proliferation assay was realized following the declaration of the manufacturer and the BrdU binding in cells was measured via the absorption A at a wavelength of 370 nm. The assay was performed as triplicate (*n*= 3) to determine the arithmetic mean of the cell proliferation rate for each combination (*n*= 4 per measurement approach). The data evaluation was carried out using concentration-effect analyses with respect to the IC_50_ (half-maximal inhibitory concentration) as a limiting parameter for basal toxicity. For this purpose, all values were normalized to the medium control in a first step, followed by non-linear regression analyses with the four-parameter logistic model (4PL/Hill function) (GraphPad Prism, GraphPad Software Inc. USA) [57,58]. For the graphical interpretation of the concentration-effect analyses, the half-maximal inhibitory effect (50% decreased threshold of medium control) was chosen preferably as correlation parameter to the IC_50_ (see Fig. 3).

#### 2.5.2 Cell vitality assay

To determine the impact of keratin particles on the vitality of NHDF and NHEK the CellTiter-Glo Luminescent Cell Viability assay (Promega GmbH, Germany) was used. Based on the quantification of the ATP presence, the number of viable cells was analyzed. For the assay, 100 µL suspension of each cell line, with a concentration of 3 × 10^3^ cells/mL, were applied in each six wells of a 96-well plate. Afterwards, the NHDF were incubated for 72 h and the NHEK for 96 h at 37°C and 5% CO_2_. All assays were carried out as individual triplicates (*n* = 3). The physiological impact of the keratin particles on the cells applied was investigated after 24 h and 48 h incubation of both the solid keratin particles and colloidal dispersion in concentrations of 0.1%, 0.005%, 0.01%, 0.005% and 0.001%. After the keratin treatment, the dispersion was removed from the cells and 100 µL of fresh cell medium of each cell line were applied. For the assay, 100 µL of the luminescence detection solution were added to the cell suspension per well and subsequently incubated for 2 min during shaking. To stabilize the luminescence signal of the assays, the plates were left at room temperature for 10 min. Finally, the vitality of cells per trial condition was ascertained based on the detected ATP level via the relative luminescence (RLU). The assay was performed as triplicate (*n*= 3) to determine the arithmetic mean of the cell proliferation rate for each combination (*n*= 4 per measurement approach). The data evaluation was carried out as described before.

#### 2.5.3 ex vivo Hen’s Egg Test on the Chorioallantoic Membrane (HET-CAM) assay

With the aim to check their mucosal tolerability, the keratin particles were tested on the intact and complex eucaryotic tissue of chorioallantoic membranes of *Gallus gallus domesticus* embryos (breed: New Hampshire). For these Hen's Egg Tests on the Chorioallantoic Membrane (HET-CAM), the eggs were incubated at 37°C and 55% relative humidity for approximately eight days. Then, the eggs were opened, and the chorioallantoic membranes of the embryos were prepared. The test compounds were a 2.88 mg/mL colloidal keratin dispersion and pure solid keratin particles. Each 200 µL of the colloidal dispersion and 10 mg of the powder were applied to the highly vascularized chorioallantoic membrane of the embryos, and alternations as bleeding, coagulation, and/or vascular changes were observed for five minutes and documented. As negative control substance *Aqua ad injectabilia* was used with the aim to cause no effects. For the positive control, an aqueous solution of 1% SDS (Carl Roth GmbH & Co. KG, Germany) was used to induce an effect. The quantification of the tolerability was ascertained based on a visual score of CAM toxicity parameters according to [59]. To evaluate the irritation potential of the substances, each test item was conducted on *n*= 6 different chorioallantoic membranes of hen’s embryos [59].

#### 2.5.4 Control of microbiological state

Microbiological smears were carried out to control the microbiological state of the extracted keratins to ensure the microbiological safety of the product. The investigation was conducted in accordance with the currently valid test standard: the European Pharmacopoeia (Ph. Eur.) 6.0 (6.0/2.06.12.00). In each case, 1 g of the keratin particles was dissolved in 9 mL of NaCl peptone buffer solution (Sigma-Aldrich), after which 1 mL was plated in duplicates (*n*= 2) in a 1:10 or 1:1000 dilution in a plate casting process on soy peptone-casein peptone medium and incubated aerobically at 30°C for 5 days (TAMC) or on SGA chloramphenicol and incubated aerobically at 22°C for 7 days (TYMC) and then evaluated.

## 3 Results

### 3.1 Raw material and extraction process

In a pilot test, different raw materials, e.g., human hair, sheep wool, snakeskin and chicken feathers were tested as possible source material (for details see section S1.1 in the supplemental information). From this, chicken feathers were found to be the best source of raw material with respect to the product desired. Moreover, different extraction procedures applying toxic, mutagenic, carcinogenic or irritating substances and final precipitation were compared in a literature-based work to the procedure presented (see section S1.2 in the supplemental information). Finally, a sustainable extraction method of keratins with a yield of 40 % was found.

### 3.2 Protein biochemical characterization

A preceding 1D SDS-PAGE study revealed two main protein fractions of the keratins extracted with about 10 kDa and 23 kDa (see Fig.S2 in the supplemental information, section S2). With the aim to get a closer look into the protein sequences extracted and to differentiate more detailed between then, a 2D SDS-PAGE (see Fig.S3 in the supplemental information, section S3) was performed, where the precise separation of the protein fractions and the determination of the isoelectric points (pIs) were realized. Here, a main fraction of around 10 kDa was detected with a pI range of 3 - 5, as well as secondary fractions of 15-25 kDa (pI 3-4), 25-35 kDa (pI 3-3.5) and even < 10 kDa (pI 7-9). By mass spectrometry, one selected spot from the gel was identified as feather keratin with a mass of about 10 kDa and a pI of 3.5 - 4. More details about spots detected are presented in the supplemental information, section S3.

Samples were analyzed with mass spectrometry, whereby the proteins were extracted from the 2D SDS-PAGE gel spots. The resulting peptides were of non-standard size and contained multiple missed cleavages. However, this was compensated within the search engine algorithm (Byonic, v5.1.1 – Protein Metrics Inc, USA). Peptide-spectral matches were subsequently manually curated. One 2D SDS-PAGE spot (ID 4499) was identified as feather keratin (4 P20308-CFP) with a coverage of 60% and 26 unique peptides (see Table S4 in the supplemental information, section S4). From the MS data, the feather keratins had 98 amino acids and a theoretical pI of 7.62. Here, feather keratins 1, 2, 3, and 4 were identified next to beta-keratin. Thereby, feather keratin 3 was the main fraction with a proportion of about 36% (see Figure S5). Details about the other keratin-related or from feathers extracted proteins identified as well as a statement regarding their quantitative ratios are listed and discussed in the supplemental information (sections S4 and S5).

### 3.3 Biophysical characterization

**Fig. 1:**
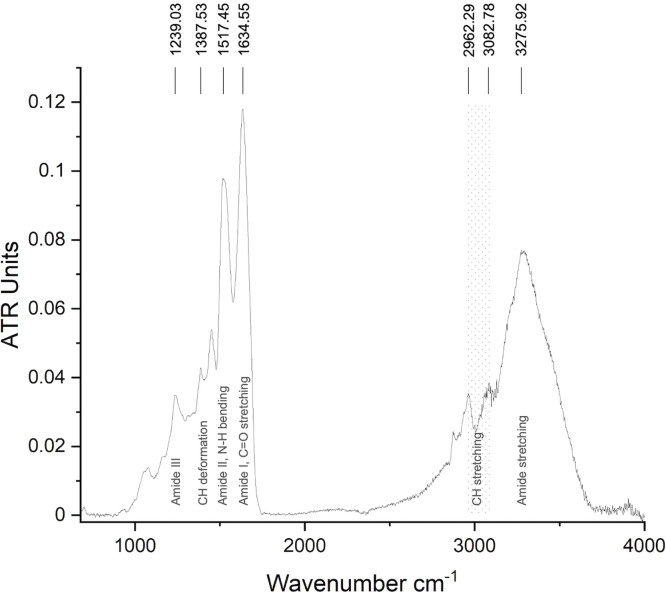
Overview of FT-IR spectra (sum of *n* = 5) of keratins. The FT-IR spectroscopy was carried out as attenuated total reflection under use of ZnSe crystal with 20 mm diameter and 45° incidence angle. Altogether *n* = 32 scans per sample were realized.

The FT-IR spectroscopy was used to ascertain the specific spectrum of the feather keratins (Fig. 1). For the protein powder, an amide III peak was determined at 1239 cm^-1^. Furthermore, the N-H bond of the amide II was documented at 1517 cm^-1^, as well as the strong C=O stretching of the amide I at 1634 cm^-1^. Finally, the CH_3_ stretching between 2962 cm^-1^ – 3082.78 cm^-1^ and the amide stretching of O-H and N-H at 3275 cm^-1^ were detected.

The DLS measurements were carried out to reveal the size of the keratin particles in colloidal dispersion. Two main fractions were detected applying the 173° measuring angle in the protein dispersion with a PDI of 0.414 ± 0.045 (see Table S6 in the supplemental information, section S6). One fraction possessed a particle size from an average of 47 nm ± 10 nm and an amount of 8%. The higher quantitative ratio of 92% was determined for particles with a size of approximately 299 nm ± 52 nm.

To analyze the charge of the keratins, electrophoretic light scattering was carried out. The colloidal keratin dispersion showed a zeta potential from an average of -25.0 mV ± 2.6 mV (dispersed in 10 mM NaCl solution) or -40.4 mV ± 3.9 mV (dispersed in DI water) and therewith a negative charge of the keratin particles. In the scope of the pH titration from 3 to 10 an isoelectric point of 3.2 was ascertained for the keratin dispersion (zeta potential = 0 mV) and a particle size of 200 µm ± 42 µm (see Fig. S6 in the supplemental information, section S6).

Using a SEC-MALLS device, an adequate separation of the expected fractions could not be achieved on any of the columns and constant flow rates used, as Figure S7 (A, B, C, and D in the supplemental information, section S7) is presenting. Reducing the sample concentration from 10 mg/mL to 1 mg/mL resulted in minimal improvement of the peak separation (data not shown). Furthermore, the RI detector did not provide sufficient signal intensity for the determination of the *dn/dc* value for the two fractions eluting first. This would have been necessary, however, as no values can be found in the literature for the refractive index increment of keratins in aqueous dispersion. A combination of two TSKgel G2500PW_XL_ columns in a row showed no significant benefits regarding the resolution (Figure S7A).

### 3.4 Microscopic characterization

**Fig. 2:**
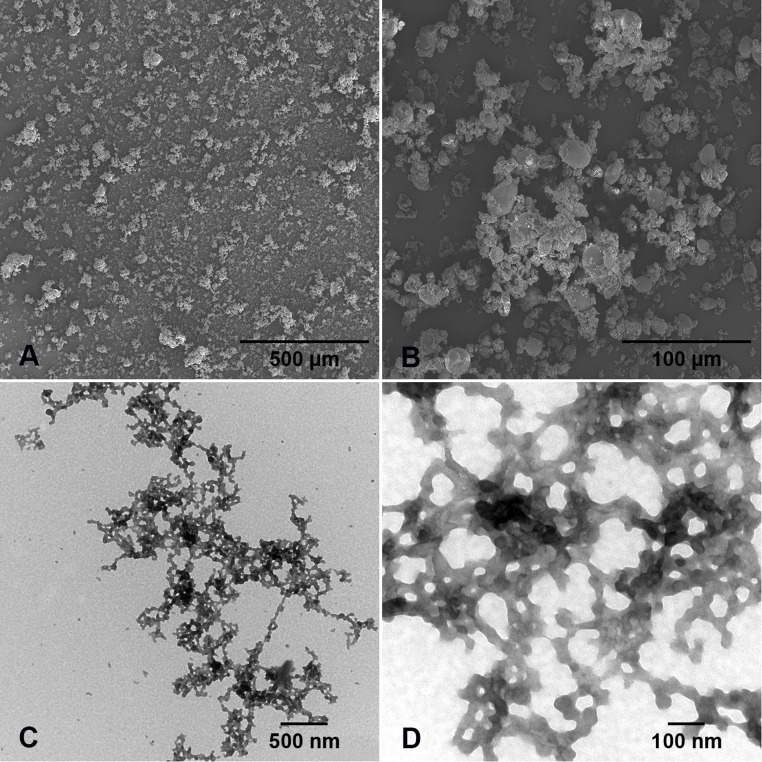
A, B: SEM images of keratin powder. The particle size and shape of the keratin powder was ascertained in SEM at 5 kV (measured particles from Fig. 2B *n* = 100, sum of spray drying batches *n* = 2). C, D: TEM images of colloidal keratin particles. To ascertain the particle size and shape, a 0.5 mg/mL colloidal keratin dispersion was applied on a TEM grid, which was contrasted with a 1% uranyl acetate dispersion. (C) Aggregates of keratin particles. (D) Detailed image of congregated keratin particles of which *n* = 25 were measured.

To characterize the size and shape of the solid keratin particles and in colloidal dispersion, microscopic investigations were performed. The keratin powder was analyzed by SEM examinations. Altogether, 80% - 90% of the particles were filaments with a size of about 5 - 30 µm (Fig. 2 A). The average particle size after the spray drying amounted reproducibly to 15 µm ± 6 µm (*n*= 100, Fig. 2 B). Approximately 10% to 20% of the keratin powder composed of aggregates from an average size of 30 µm to a maximum of 250 µm.

The TEM studies were carried out to analyze the physical appearance of the keratin particles in the colloidal dispersion. Within the protein dispersion, the particles exhibited a mainly uniform morphology and a filament structure (Fig. 2 C). Conditioned by the aggregation behavior of the particles during their application on TEM grid, the determination of their size was difficult. The approximate particle size of individual filaments was 71 nm ± 24 nm (*n*= 20; Fig. 2 D).

### 3.5 Characterization of physiological compatibility

**Fig. 3:**
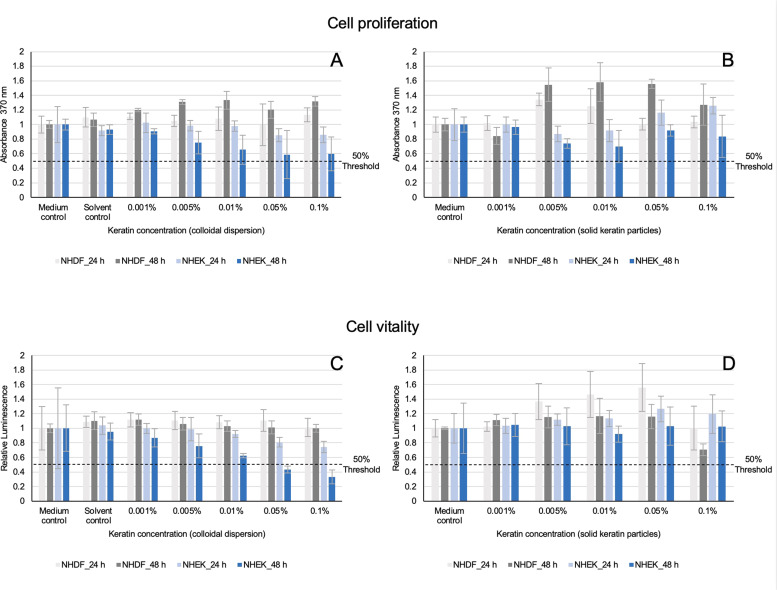
Top line (A and B): Cell proliferation rates of NHDF (grey) and NHEK (blue) cells after keratin application for 24 h (bright-colored) and 48 h in concentrations of 0.001%, 0.005%, 0.01%, 0.05%, and 0.1%. The proliferation rates of the cells were determined through the BrdU incorporation during DNA synthesis at an absorbance A of 370 nm. (A) BrdU amount in cells after incubation with colloidal keratin dispersion. (B) BrdU amount in cells after incubation with solid keratin particles. Bottom line (C and D): Cell vitality rates of NHDF (grey) and NHEK (blue) cells after keratin application for 24 h (bright-colored) and 48 h in concentrations of 0.001%, 0.005%, 0.01%, 0.05% and 0.1%. The vitality rates of the cells were determined through the ATP presence after addition of the CellTiter-Glo Luminescent (Promega). (C) ATP amount in cells after incubation with colloidal keratin dispersion. (D) ATP amount in cells after incubation with solid keratin particles. Both lines: All control cells were cultivated in their medium without keratin addition. To determine the cell proliferation rate and cell vitality for each combination, the tests were performed as independent triplicates *n* = 3 with *n* = 4 per measurement approach. The 50% threshold lines label the half-maximum inhibitory effect of the medium control, correlating to an effect caused by an IC_50_.

The BrdU assay was utilized to ascertain the proliferation rates of the NHDF and NHEK following an incubation period of 24 h and 48 h with the colloidal keratin dispersion and the solid keratin particles, respectively. Overall, none of the keratin concentrations caused cytotoxic effects beneath the 50 % effect threshold in both basal cell lines at any time, so that the IC_50_ can be concluded to be > 0.1 % keratin concentration. In detail, for the NHDF, no reduction of the proliferation rate could be detected to both time points, neither for the colloidal keratin dispersion nor for the solid keratin particles (Fig. 3A and B). The application of keratin particles resulted in elevated levels of cell proliferation. The addition of colloidal keratin dispersion caused an increase of cell proliferation up to 33% ± 12% (concentration 0.01%, Absorption A= 1.33, 48 h, Fig. 3A) and the application of the keratin powder effected an enhancement of up to 60% ± 27% (concentration 0.01%, A= 1.58, 48 h, Fig. 3B) compared to the medium control (A= 1.0, 48 h, Fig. 3A and B). In contrast, the NHEK showed a reduction in their proliferation rates after the application of the colloidal keratin dispersion (Fig. 3A). After 24 h, the cell proliferation rates decreased for keratin concentrations of 0.05% and 0.1% (A=0.85, 24 h, Fig. 3A). Especially, the keratin treatment of the NHEK after 48 h caused a higher reduction of the proliferation rate after the addition of colloidal keratin dispersion (Fig. 3A). Thus, the cell proliferation rate decreased concentration-dependent resulting in an average reduction from 25% ± 15% (concentration 0.005%, A=0.75, 48h, Fig. 3A) up to 40% ± 22% (concentration 0.1%, A=0.59, 48 h, Fig. 3A). After the incubation with solid keratin powder, a declining trend of the NHEK proliferation behavior was detected after 24 h caused by the applied concentrations of 0.005% and 0.01% as well (Fig. 3B). All other tested keratin concentrations showed similar or (slightly) increased cell proliferation rates after 24 h of treatment with keratin powder (Fig. 3B). An incubation period of 48 h with solid keratin powder revealed comparable proliferation activities of the NHEK (Fig. 3B). But the cell proliferation decreased noticeably by around 30% ± 20% after the addition of 0.005% (A=0.74) and 0.01% (A= 0.70) solid keratin particles and increased again for the higher concentrations applied (Fig. 3B).

The vitalities of the NHDF and NHEK cells were ascertained by the determination of the ATP presence in the cells after the application of both colloidal keratin dispersion or solid keratin powder particles for 24 h and 48 h (Fig. 3C and D). Except of two, none of the tested keratin concentrations elicited cytotoxicity in either basal cell line at any time point (Fig. 3C and D). Only for the NHEK after 48 h a clear concentration-effect relationship was observed for the keratin concentrations in colloidal dispersion of 0.05% and 0.1%, where the limit of the half-maximum inhibitory effect of the medium control was exceeded as a result of a concentration-dependent effect (keratin concentration of 0.001% (relative luminescence (RLU) =0.87) to 0.1% with a NHEK cell vitality reduction of 67% ± 9% (RLU= 0.3) (Fig. 3C). In detail, the determination of the ATP presence in the NHEK cells showed decreasing effects on the cell vitality after the application of the colloidal particles (Fig. 3C). Already after 24 h of keratin treatment with 0.1% colloidal particles, the vitality was reduced by 26% ± 8% (RLU=0.74, Fig. 3 C). For the NHDF cells supporting effects of colloidal keratins were observed with respect to their cell vitalities (Fig. 3C).

The solid keratin particles only decreased the rate of cell vitality in a concentration of 0.1% after 48 h in NHDF cells about 30% ± 8% (RLU= 0.70, 48 h, Fig. 3D). For all other concentrations and time points, improvements on the NHDF cell vitality rate were noted, for example an increase of 56% ± 32% (concentration 0.05%, RLU=1.56, 24 h, Fig. 3D) compared to the medium control (RLU=1.0). In contrast to the colloidal dispersion, the application of the solid keratin particles affected no reduction of the NHEK cell vitality (Fig. 3D). After 24 h, an increase of cell vitality up to 19% ± 26% (concentration 0.01%, RLU=1.19, 24 h, Fig. 3D) compared to the medium control was visible.

The HET-CAM investigations were used to evaluate the mucosal tolerability of the feather keratin particles in powder form and as colloidal dispersion in DI water. No effects of hemorrhage, vascular lysis, and/or coagulation were observed on the chorioallantoic membranes for the keratin-containing test compounds (Table S8 in the supplemental information, section S8). In contrast, the used positive control, 1% SDS, provoked a strong irritative effect on the chorioallantoic membrane with an irritation score of 9.80 ± 0.65 whereby no irritative potential was observed for the negative control, *Aqua ad injectionem*, on the chorioallantoic membrane (Table S8 in the supplemental information).

Microbiological testing of the pure keratin particles in powder form as a non-sterile product revealed a total aerobic microbacterial count (TAMC) of 1.7 × 10^2^ CFU/g, which was considered as harmless, as a limit value of < 10^3^ CFU/g was not reached (see Table S9 in the supplemental information, section S9). The value for the total yeast and mold count (TYMC) with 10 CFU/g was as well beneath the limit value of 10^2^ CFU/g.

## 4 Discussion

One of the primary goals of biomaterial research is to develop biodegradable polymers for applications in cosmetic products, pharmaceuticals, and as biomedical agents. Over the last few decades, keratins have emerged as a valuable and underutilized source among other available biomaterials. The proteins are major components of hair, wool, nails, hooves, feathers and horns of different animals and mammalians, and are a waste product of the manufacturing industry. But, from the regulatory point of view, human raw materials are forbidden for application in cosmetics following European law [60]. As a uniform production process for cosmetic, pharmaceutical and *in vitro* applications was intended in the study presented to ensure the reproducibility and to reduce production costs, human hair could not be used for further keratin extraction processes. Due to allergenic potential of other hair sources, as from dogs and cats (Can f1, Can f2), hairs in total were avoided. The lacking sources and quantities of snakeskin made this material unusable as well. Finally, alongside wool, using waste from chicken poultry figured out to be a worthwhile reusage of the biomaterials for keratin powder production. One of the main reasons why chicken feathers are considered as promising future biomaterial and natural protein source, is that with 65 million tons of feathers are available as slaughterhouse waste worldwide every year. Furthermore, internal preliminary investigations on other keratin sources (wool, horn, feather meal) identified feathers, especially chicken feathers, as the most suitable source of raw material with a sufficient yield of keratins. Based on this, the aim of the study was (a) to find an environmentally friendly and sustainable production of full-sequenced keratins, (b) to characterize the product comprehensively and (c) to introduce the keratin particles as biological material with a perspective for the external application on skin or model substance for damaged SC.

Currently, feather keratin products are commercially available either in powder form or as colloidal dispersion. But, due to microbial, acidic or basic hydrolysis, they are degraded into small peptide structures, meaning they are no longer intact, complete keratin proteins [45,61]. In addition, toxic and carcinogenic substances,such as 2-mercaptoethanol and thiourea, were used in the keratins´ extraction processes [48]. Moreover, SDS was used in comparable extraction methods found in the literature (see section S1.2 in the supplemental information), which is known to be irritative. Here, for instance, SDS was even used as positive control in the HET-CAM study performed. But, as our intention was to receive a non-irritative, non-toxic product, we presented an extraction procedure using substances that were harmless to health and were able to extract natural and, above all, intact keratin proteins. Due to the chemical agents and extraction conditions chosen, the keratins could be received as a powder with a beneficial degree of water-solubility. Here, a special mixture of urea, L-cysteine, and Tris-HCl was used in the basic pH of 10.5. In detail, in a concentration of 10 M, urea induced the swelling of the proteins sequences by breaking off the hydrogen bonds [62,63]. The reducing effect of L-cysteine caused the breaking of the intramolecular disulfide bonds, which finally facilitated the extraction of the bio-active proteins with intact primary and secondary structures. Indeed, the authors see potential to adapt parameters of the extraction procedure to facilitate more optimized and process-controlled keratin particles, regarding their e.g. particle size, molecular mass or aggregation behavior as discussed in detail at the appropriate place.

For identifying the keratins, the FT-IR revealed absorption bands caused by peptide bonds (-CONH-), where the amide I correlated vibrational bands for C=O stretching at 1634 cm^-1^ was, as expected, between 1690-1600 cm^-1^ [64]. Amide II should occur in the range of 1580 – 1480 cm^-1^ [64], where the determined N-H bending of 1517 cm^-1^ was found. The Amide III – peak (1239 cm^-1^) into the range of 1300-1220 cm^-1^, demonstrating C-N stretching and C=O bending vibration [64]. Moreover, the conformations were identified as β- sheets (1630 cm^-1^) for amide I at 1634 cm^-1^, dominating over α- helices (random coil) at 1660 cm^-1^. For amide II, α- helices (1540 cm^-1^) were again dominated by β- sheets (1520 cm^-1^), present at 1517 cm^-1^. For amide III, typical bands were known (α- helices at 1235 cm^-1^, β- sheets at 1265 cm^-1^), which were here (1239 cm^-1^) representing random coiled structures [65]. From that, beta-keratin could be identified as the main fraction of the received keratins with α- helical structures besides. Previous works described the α- conformation predominantly (∼ 66%) over the β- sheets (∼ 33%) [17]. However, the FT-IR results are limited regarding the conformational rearrangements of the keratins, which could be influenced by the particles´ drying procedure or urea residues. Here, complementary solution-state techniques as circular dichroism could help to clarify the findings as part of future work. The 2D gel electrophoresis approach identified a main fraction of the keratin dispersion with about 10 kDa of molecular mass. This is congruent with calculated values and results found in the literature, where about 10-14 kDa were described for keratin proteins [17,66,67]. Higher weighted fractions of about 23 kDa or 50 kDa were found in some literature [43] but not further discussed or even representing the main fraction, e.g. in a work of *Yin et al*., who described a single fraction of about 20 kDa [68]. Here, we have distinct evidence, that the 23 kDa fraction is attributed to other keratins extracted (see Table S4 in the supplemental information) but no feather keratins. From the MS data, keratins were clearly identified only in the 10 kDa fraction with sufficient proof (*p* < 0.001). In order to get an idea about the proportions of the keratins extracted, semi-quantitative analyses based on the MS results were performed, which revealed feather keratins 1-4 to be in sum about 65% of the keratin particles (see Fig.S5 in the supplemental information). Besides beta-keratin (with a ratio of 14%), only 20% of the particles were not identified as keratins. For further characterization of the feather keratin particles produced, the pI was calculated by the MS to be 7.62, but following the electrophoresis results in the 2D gel, the pI of keratin was found to be between 3.5 and 4. Depending on the kind of refolding, the dimerization could provide different positions of the charges, so that for the dimer a different pI could be determined here compared to the theoretically values. At this point, we focused on the pI of 3.5, which is comparable to the results we received from the titration investigation per DLS with a pI of 3.2. As for keratin hydrolysate a pI of 4.4 was described [69], which is, compared to our result, a further distinct evidence for the presence of the full protein structure here.

Using a DLS device, the particle size of keratin in colloidal aqueous dispersion was found to be of approximately 299 nm ± 52 nm, whereby TEM presented 71 nm ± 24 nm of molecular diameter for the keratins. A reason for this discrepancy can be the agglomeration behavior of the keratin particles, the methodology or the kind of keratin production: Depending on the way of drying and further preparation, the size of the solid powder keratin particles can differ. Applying lyophilization of the colloidal dispersion, *Fujii and Li* found their β- keratin particles to have a diameter of about 0.5 – 3 µm [43], what was in accordance to the preparation of the aspired films. As the SEM results revealed, a future optimization of the drying procedure and post-drying production processes could help to facilitate homogeneous and size-controlled particles, which will be necessary for further technological applications. To evaluate the agglomeration tendency of the keratin particles, the zeta potential was determined. With about -40.4 mV (in aqueous dispersion), meaning a negatively charged outer surface of the keratins, the keratin dispersion was titrated from pH 3 to 10, were an isoelectric point of 3.2 was observed. With further decreasing pH values, the particle size increased enormously due to an intensive agglomeration behavior of the keratin filaments. This phenomenon was reversible increasing the pH above 3.2 again. Indeed, the DLS method could reveal false-positive results due to the filament structure of the keratins, for instance as the particles were hindered in their movement during the measurement and were therefore detected as larger than they actually were. From that, worthful hints about the surface characteristics of the keratins were received, which are necessary, e.g. for their implementation in skin models or skin treatment.

As no values of the refractive index for feather keratins in aqueous dispersion are known from the literature, the determination of the molecular weight via SEC-MALLS was limited as the particle size was to close at the cut-off-point. Only a work of *Ma et al*. presented comparable size exclusion chromatographical results, but with the same limit of peak separation [70]. Thus, they characterized hydrolyzed keratin peptides from NMMO (*N*-methylmorpholine *N*-oxide) extraction and proceed no peak integration and missed to discuss the problem of peak separation. However, from that point, we cannot refer a defined molecular weight from the presented approach.

In the cell culture assays, for keratin in colloidal dispersions in concentrations of 0.05% and 0.1%, after 48 h the detected cell vitality was decreased beneath the 50% threshold effect level (half-maximum inhibitory effect), whereby both can be interpreted as concentrations higher than a correlated IC_50_. But it has to be considered, that for both concentrations after 48 h a swelling of the keratins was observed, which seemed to act as physical barrier to the luminescent agent. From this, we cannot exclude, that beneath the physical barrier the remained cells were still vital but not detectable as active, as no penetration of the luminescent agent into the cells was possible anymore. Here, false-negative results have to be considered. In general, based on the results of cell culture assays on NHDF and NHEK cells, the use of keratin both as solid particles and in colloidal dispersion can be considered as safe following the OECD guidance document [57], as effects beneath the 50% threshold, which would correlate to the IC_50_ level, were not reached in a majority. Ultimately, however, clinical application data have to prove the safety of keratin applied onto the skin. From the microbiological tests and the application of the keratin particles to *ex vivo* eukaryotic tissue of the endothelial and epithelial cells of the chorioallantoic membrane in the HET-CAM study, the keratin particles revealed neither irritative potential on sensible membranes nor any safety issues regarding microbiological infestation. This was of high benefit for the intended application as a model substance for keratolytic skin and even as supplemental and supportive material in the treatment of damaged SC. Finally, the idea of using keratin particles in biofilms for wound healing or temporarily replacing SC components such as keratins together with lipids in diseased SC can be pursued further.

## 5 Conclusion

Within the last decade, the reuse of waste, especially when it is ubiquitously available, and its transformation into a new usable material is of great interest in terms of sustainability. In the study presented, an intact full-sequenced keratin in powder form could be extracted environmentally friendly from chicken feathers due to the absence of toxic extraction agents. However, the yield was smaller compared to extraction methods applying SDS, but the irritative potential of SDS led us to the decision, to avoid its usage. Due to this, a non-irritative product was received, as the HET-CAM study revealed. Moreover, based on the eukaryotic cell culture assays performed, we have no distinct hint about safety issues correlated to the keratin particles. This was of enormous benefit for the intended application on irritated or damaged skin as humectant in cosmetics or excipient in pharmaceutics. The harmless keratin particles were furthermore identified as mainly feather keratins by MS, whereby FT-IR technique revealed intact primary and secondary protein structures. The solid keratin powder particles were soluble in water sufficiently for the intended application and could agglomerate below the pI of the keratin. Besides, ongoing characterization with respect to effects of the extraction procedure to the protein rearrangement and the optimization of the extraction process itself with a focus on the quantitative process-structure-property relationship need attention as part of future work. Moreover, the kind of bioactivity and the introduction of the keratin powder as model substance to *in vitro* skin experiments or as excipient in dermal applications could reveal the performance of the keratins as medical biomolecule.

## Funding sources

This research did not receive any specific grant from funding agencies in the public, commercial, or not-for-profit sectors.

## CRediT authorship contribution statement

**Julia Chuttke:** Writing – original draft, Validation, Investigation, Formal analysis, Data curation. **Luisa Scholz:** Writing – original draft, Investigation, Formal analysis, Data curation. **Johannes Wohlrab:** Resources, Methodology, Funding acquisition, Conceptualization. **Mandy Koch:** Writing – original draft, Validation, Investigation, Formal analysis, Data curation. **Gerd Hause:** Writing – original draft, Validation, Investigation, Formal analysis, Data curation. **Matthew Fuszard:** Writing – original draft, Validation, Investigation, Formal analysis, Data curation. **Adina Eichner:** Writing – original draft, Supervision, Project administration, Methodology, Conceptualization.

## Declaration of competing interest

The authors declare the following financial interests/personal relationships which may be considered as potential competing interests:

JW declares, that he has received honoraria for consulting and/or lectures, and/or sponsoring for scientific projects and/or clinical studies from the following companies in the last five years: Abbvie, ACA, Actelion, Almirall, Apogee, BayPharma, Beiersdorf, Bioderma, Biogen, BMS, Boehringer Ingelheim, Celltrion, Dermapharm, Evolva, Galderma, GSK, Hexal, Infectopharm, Incyte, Janssen-Cilag, Johnson & Johnson, Klinge, Leo, Lilly, L‘Oréal, Medac, Medice, Mibe, MSD, Mylan, Novartis, Pierre Fabre, Pfizer, Regeneron, Rigi, Sanofi-Genzyme, Skinomics, UCB and Wolff. AE has received remuneration from Novartis over the past 2 years as part of a scientific project unrelated to this manuscript. JC, LS, MK, MF and GH declare no known competing financial interests or personal relationships.

## Data Availability

Data will be made available on request.

## Glossary

DLS: Dynamic Light Scattering, laser-based particle size determination in fluids
FT-IR: Fourier Transformation Infrared Spectroscopy, via an interferometer structure elucidation of substances are possible next to their qualitative and quantitative determination
HET-CAM: Hen’s Egg Test on the Chorioallantoic Membrane (HET-CAM) assay, an *in vitro* method for testing the mucosal tolerance of chemicals on chorioallantoic membrane of chicken embryos.
MS: Mass Spectrometry, generation, separation, and recording of ions from organic or inorganic substances according to their mass and charge
NHDF: Normal Human Dermal Fibroblasts are primary eukaryotic cells applied in cell culture assays.
NHEK: Normal Human Epidermal Keratinocytes are primary eukaryotic cells applied in cell culture assays.
SEC-MALLS: Size Exclusion Chromatography coupled with Multi Angle Light scattering
SEM: Scanning Electron Microscopy, imaging technique, based on the interactions of electrons and the object to be investigated
SDS-PAGE: Sodium Dodecyl Sulfate – Polyacrylamide Gel Electrophoresis, Separation of protein structures alongside their particle size
2D SDS-PAGE: Two-dimensional SDS-PAGE, Separation of protein structures alongside their charge and their molecular
TEM: Transmission Electron Microscopy, imaging technique, that visualizes the transmission of electrons through a thin material film

## Appendix Supplementary materials

Image, application 1
