# Supplementary material for: Extraction of keratin particles as intact protein sequences from chicken feathers and their characterization
Source: Biomater Biosyst. 2026 Jan 17;21:100128. doi: 10.1016/j.bbiosy.2026.100128 (PMC12861210; doi:10.1016/j.bbiosy.2026.100128)
Supplement: Supplementary file 1 [file mmc1.pdf]

Supplemental information

Extraction of keratin particles as intact protein sequences from chicken feathers and their characterization

Julia Chuttke[a,b*], Luisa Scholz[a*], Johannes Wohlrab[a,b], Mandy Koch[c], Gerd Hause[d], Matthew Fuszard[e], Adina Eichner[a,b]

[a] Department of Dermatology and Venereology, Martin Luther University Halle-Wittenberg, Ernst-Grube-Str. 40, 06120 Halle, Germany

[b] Institute of Applied Dermatopharmacy at Martin Luther University Halle-Wittenberg, Weinbergweg 23, 06120 Halle, Germany

[c] Institute of Chemistry, Food Chemistry, Martin Luther University Halle-Wittenberg, Kurt-Mothes-Str. 2, 06120 Halle, Germany

[d] Microscopy Unit, Biocenter, Martin Luther University Halle-Wittenberg, Weinbergweg 22, 06120 Halle, Germany

[e] Interfaculty Core Facility – Proteomic Mass Spectrometry, Charles Tanford Protein Center, Martin Luther University Halle-Wittenberg, Kurt-Mothes-Str. 3a, Halle, Germany

* These authors contributed equally to this work.

Corresponding author:

Adina Eichner, PhD
Department of Dermatology and Venereology
Martin Luther University Halle-Wittenberg
Ernst-Grube-Str. 40
06120 Halle (Saale). Germany
+49 345 557 3819
adina.eichner@medizin.uni-halle.de

S1

Pilot test including choice of raw material and optimization of extraction procedure

1. Choice of raw material

To find an optimal raw material for the keratin extraction, several materials were included into the evaluation. Here, the following points were included in the survey proceeded for the primary purpose: Availability of the raw material, sustainability due to the material usage, yield of the extracted keratin and regulatory aspects. The outcome is summarized in Table S1.1. To compare the yield of the raw material, the extraction process was continuously unique, like presented in the manuscript for chicken feathers.

Material	Availability	Sustainability	Yield	Regulatory problems	disadvantages
Wool (shep)	+++	+++	31 %	Existing patents	Allergenic potential
Hair (human)	+++	+++	39 %	EU regulation No. (EC) 1223/2009 prohibits human materials in cosmetics	/
Hair (animal)	++	+++	41 %		Allergenic potential
Feathers (chicken)	+++	+++	40 %		
Horn meal (mixture of different species)	++	+	64 %		Hydrolyzed meal with peptides
Snakeskin	- -	- -	22%		Low natural occurrence

Table S1.1: Comparison of different raw materials featuring as keratin source

With respect to the availability, the highest degree (+++) was set for sources, where the material is produced anyway. Degree of (- - -) would mean an extra murder of the animal. In case of skin from snakes, the amount of natural occurring skin from growing snakes was interpreted as too minimal for an industrial keratin production. Overall, chicken feathers revealed the most balanced product of intact protein (and not hydrolysates compared to horn meal), if the availability (due to poultry from slaughterhouses) and the absent regulatory inhibitions next to any allergic potential were taken into account.

2. Choice of extraction parameters and chemical agents

Now the extraction process presented was compared to other methods and their respective results found in the literature (Table S1.2). With the aim of a non-toxic extraction procedure, we avoided toxic, mutagenic, carcinogenic or irritating substances, e.g., 2-mercaptoethanol, thiourea, SDS or sodium metabisulphite for the keratin extraction. Even other green extraction methods used SDS and accepted its irritative potential as it was irrelevant to the application intended. The work of Zhu et *al.*, [1] and works of the group around Yiqi Yang [2, 3] described a comparable extraction buffer used for a keratin extraction from wool or duck feathers (see Table S1.2).

Besides the difference in the raw material, the temperatures during the extraction procedures were different as well. For instance, with 90 °C, the extraction product was more hydrolyzed into peptides than at 65 °C. Moreover, for the extraction buffer, urea was chosen in a concentration of 10 M, whereby from the literature it is known, that above a concentration of 8 M, urea causes a swelling of the protein structure by weakening the hydrophobic interactions between the peptides [4, 5]. Here, our procedure presented is the first one applying urea in the higher concentration than 8 M compared to the literature. Due to this, the reducing effect of L-cysteine on the intramolecular disulfide bonds was even supported. Tris-HCl ensured the basic milieu during the keratins´ extraction process. Moreover, we avoided the precipitation of the keratins by acid or salt to ensure the intact protein sequence compared to methods found in the literature [2, 3, 6, 7]. Due to this, the procedure described ensured an intact primary and secondary structure of the keratins extracted, compared to the literature, where obviously often peptides were generated. Overall, this new variant of extraction procedure ensured a non-irritating and “green” keratin product for the intended application on damaged skin.

Agent	Extraction procedures from literature						New procedure
	Xia et *al.*, 2025 [8]	Zhu et *al.*, 2025 [1]	Ma et *al.*, 2016 [6]	Xu et *al.*, 2014 [7]	Mi et *al.*, 2019 [3]	Mi et *al.*, 2020 [2]	
Reductant	Na_2S	L-cysteine	L-cysteine	L-cysteine	L-cysteine	L-cysteine, DTT	L-cysteine
c_{Urea}	5 M	8 M	8 M	8 M	8 M	8 M	10 M
Raw material	wool	wool	chicken feathers	chicken feathers	duck feathers	duck feathers	chicken feathers
Surface repulsion	SDS	--	SDS	SDS	--	--	--
pH during extraction	--	10.5	10.5	10.5	8.5	8.0	10.5
$T_{extraction}$	60 °C	90 °C	70 °C	70 °C	70 °C	70 °C	65 °C
Yield	40-50%	?	60 %	71 %	55 %	60 %	40 %
Precipitation with … at pH (x).	--	?	HCl (pH 4)	HCl (pH 4)	HCl (pH 6)	HCl (pH 4.6)	--
Use intended	Photo-responsive surface micropatterns	Biodegradeable ionotronic device	Biomedical membranes	Keratin fibers	Biomedical keratin films	Cross-linked keratin filaments by wet spinning	Humectant in cosmetics, pharmaceutical excipient, model substance for keratolytic skin

Table S1.2: Comparison of the extraction procedure proposed with examples of further procedures and their results found in the literature. The most important advantage over the other procedures presented were the absence of SDS and the lower process temperature. DTT - dithiothreitol, HCl- hydrochloric acid, SDS – sodium dodecyl sulfate

S2

<u>SDS-Polyacrylamide gel electrophoresis (SDS-PAGE)</u>

To determine the molecular weight of the keratin particles, a defined concentration of the keratin dispersion (7.5 µg/µL) was added to 2.5 µL NuPAGE LSD Sample Buffer (4x) (ThermoFisher, Darmstadt, Germany), 1 µL NuPAGE LSD Reducing Agent (10x) (ThermoFisher) and 6.5 µL bidestilled water, and denatured at 95 °C for 10 min. Always 10 µL of each prepared sample and 2 µL of marker (Spectra Low Range Multicolor, ThermoFisher) were applied on a Tricin gel (Novex 10-20 % Tricin gel, Invitrogen). The gel electrophoresis was realized at 130 V and 250 mA for 1.5 h. Afterwards the gel was transferred into a Coomassie gel stain solution (2.5 g Coomassie blue R-250, 500 mL methanol, 100 mL glacial acetic acid, 400 mL DI water) and colored overnight under shaking. Then, the gel was decolorized in a Coomassie gel destaining solution (150 mL methanol, 100 mL glacial acetic acid, 750 mL DI water), several times cleared in DI water and finally documented.

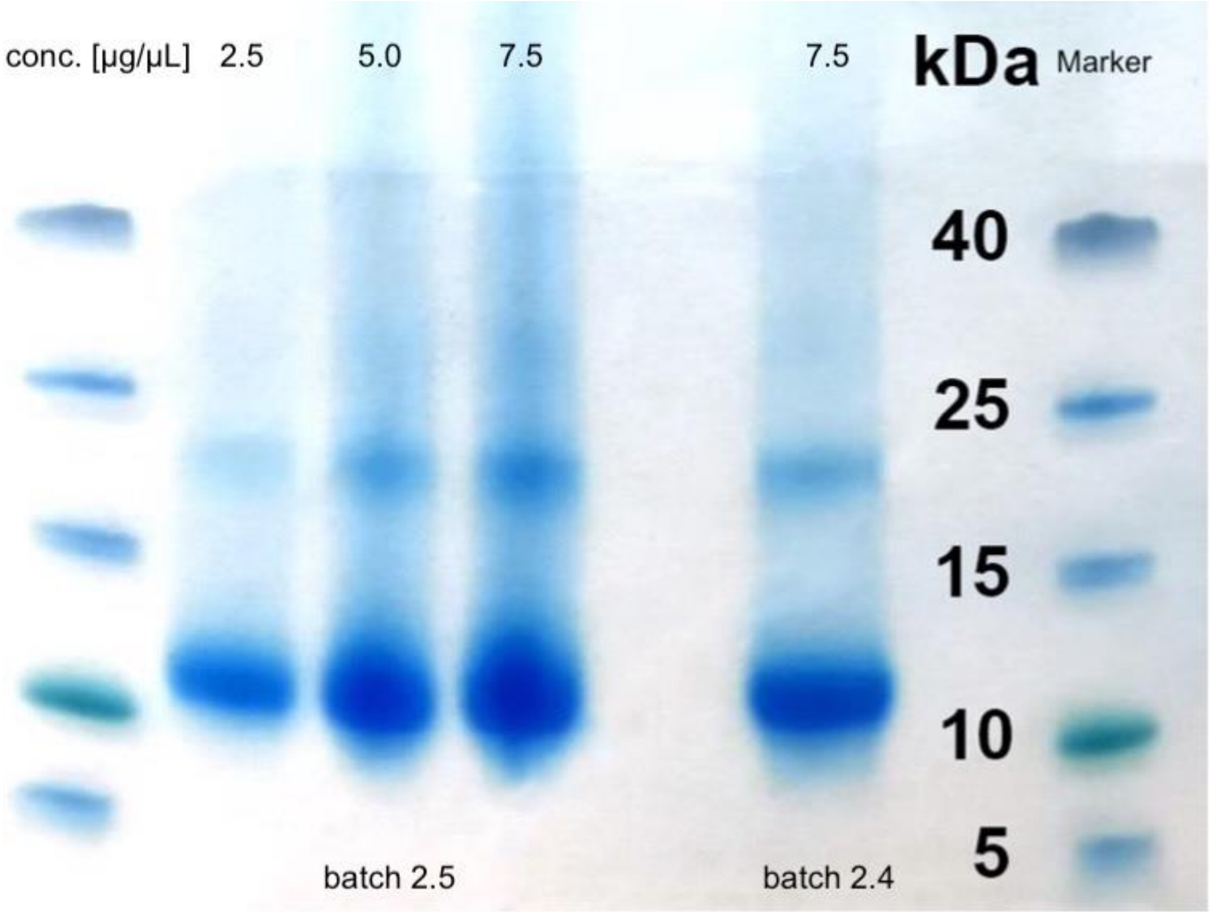

Fig. S2: Example of SDS-PAGE gel of keratins extracted from chicken feathers from two different batches and three different concentrations. A main fraction between 10-15 kDa and a minor fraction of about 23 kDa were found.

S3

2D SDS-PAGE and MS reveled the identity of the keratin spots

15 spots from the 2D SDS-PAGE pattern were picked and analyzed by MS. For spot ID 4490, feather keratin was found with a coverage of 60% and 26 unique peptides. From the 2D gel, the extracted feather keratin was found to have a mass around 10 kDa and a pI between 3.5 – 4. Moreover, keratin OS was found in ID 4490 (coverage 12-16 % and 2-3 unique peptides). In spots ID 4513 and 6220 keratin OS was found with coverages of 16-22 % and 4-5 unique peptides. The other spots, especially with masses < 10 kDa and pI values above 5 were dominated by histone and ubiquitin from chicken (*Gallus gallus*).

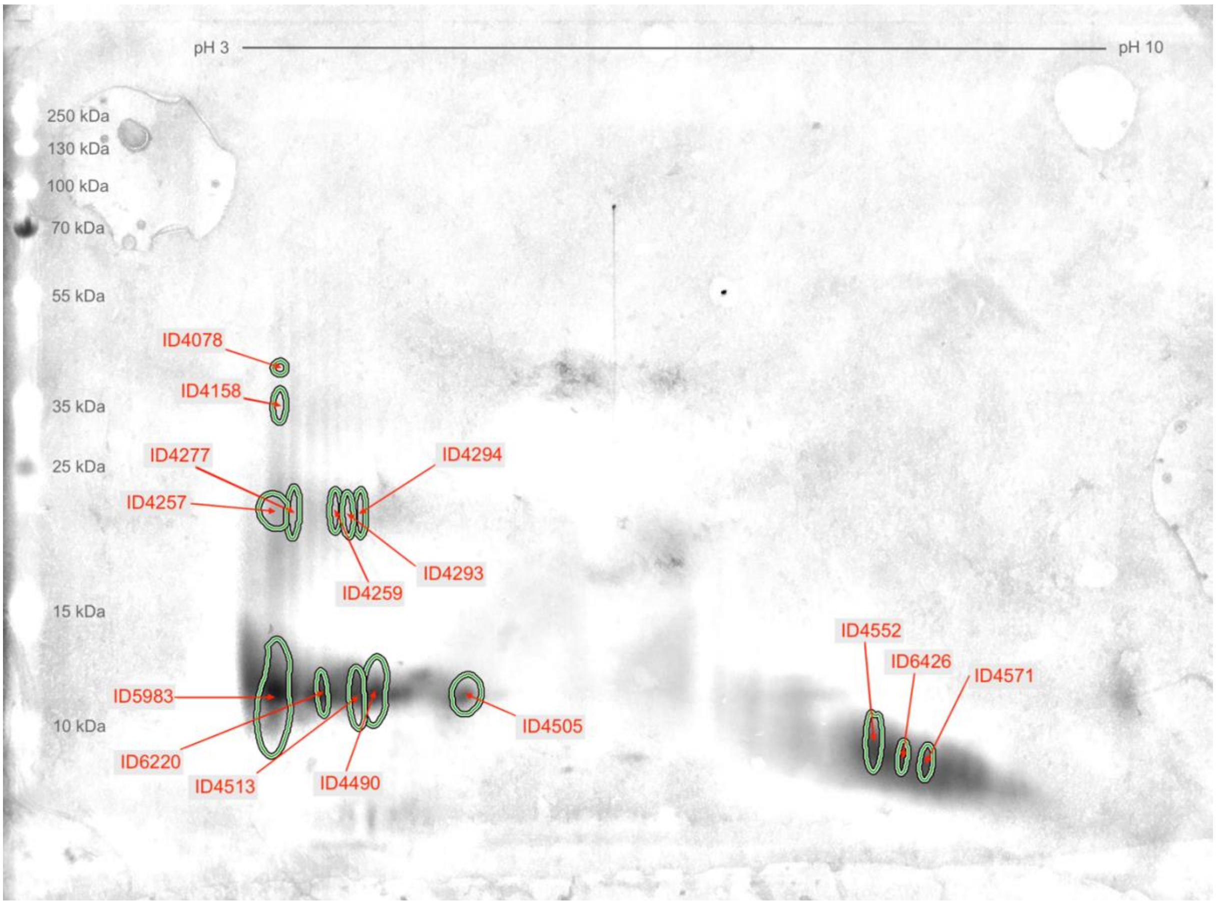

Fig. S3: 2D SDS-PAGE pattern (*n* = 1) of keratins separated by their mass and isoelectric points. All marked spots were analyzed by MS (results see Table S4).

S4

Results of mass spectrometric analyses of keratin extracted from 2D gel electrophoresis gel

see Table S4.

ID 4078	**Accession**	**Description**	**Coverage, %**	**# Peptides**	**# PSMs**	**# Unique Peptides**	**# AAs**	**MW, kDa**	**calc. pI**	**# Peptides *1**	**|Log Prob| *2**
	P0CB50	Peroxiredoxin-1 OS=Gallus gallus OX=9031 GN=PRDX1 PE=1 SV=1	11	2	2	2	199	22.3	8.1	2	4.53
	P62801	Histone H4 OS=Gallus gallus OX=9031 GN=H4-VII PE=1 SV=2	21	2	3	2	103	11.4	11.36	2	4.37
	A0A8V0Z0U1	phosphopyruvate hydratase OS=Gallus gallus OX=9031 GN=ENO1 PE=1 SV=1	6	2	2	2	445	48.7	7.06	2	4.08
	K2C1_HUMAN	(Common contaminant protein)	2	1	1	1	643	6..8	8.12	1	3.81
	P53478	Actin, cytoplasmic type 5 OS=Gallus gallus OX=9031 PE=3 SV=1	5	2	2	2	376	41.8	5.48	2	3.23
	A0A8V1A3E8	H1.10 linker histone OS=Gallus gallus OX=9031 GN=LOC121111746 PE=4 SV=1	3	1	1	1	402	41.9	10.62	1	2.63
	A0A8V0ZZM1	Tubulin alpha 1a OS=Gallus gallus OX=9031 GN=TUBA1A PE=3 SV=1	2	1	1	1	757	82.6	9.16	1	2.25
	A0A8V0YK12	ATP synthase subunit alpha OS=Gallus gallus OX=9031 GN=ATP5F1AW PE=1 SV=1	2	1	1	1	619	67.2	8.79	1	2.23
	A0A8V0ZG96	Pyruvate kinase OS=Gallus gallus OX=9031 GN=PKLR PE=1 SV=1	2	1	1	1	693	75.3	9.25	1	2.19
	P26584	High mobility group protein B2 OS=Gallus gallus OX=9031 GN=HMGB2 PE=2 SV=2	6	1	1	1	207	23.8	8.38	1	2.14
	Q5ZMJ6	ADP/ATP translocase OS=Gallus gallus OX=9031 GN=SLC25A4 PE=1 SV=1	3	1	1	1	298	32.9	9.73	1	2.09
	A0A8V0Z4J0	Ubiquitin carboxyl extension protein 80 OS=Gallus gallus OX=9031 GN=RPS27A PE=3 SV=1	4	1	1	1	233	26.3	10.15	1	2.07
	**A0A8V1A2T7**	**Keratin 18 OS=Gallus gallus OX=9031 GN=KRT18 PE=1 SV=1**	**2**	**1**	**1**	**1**	**429**	**46.9**	**5.19**	**1**	**2.05**
	Q5ZIQ3	Heterogeneous nuclear ribonucleoprotein K OS=Gallus gallus OX=9031 GN=HNRNPK PE=2 SV=1	2	1	1	1	427	47.2	6.47	1	2.04
	A0A8V0Z4Q7	Large ribosomal subunit protein eL14 OS=Gallus gallus OX=9031 GN=RPL14 PE=1 SV=1	8	1	1	1	155	18.3	10.46	1	1.95
	Q5ZLN1	Phosphoglycerate mutase 1 OS=Gallus gallus OX=9031 GN=PGAM1 PE=1 SV=3	4	1	1	1	254	28.9	7.49	1	1.95
	P84247	Histone H3.3 OS=Gallus gallus OX=9031 GN=H3-X PE=1 SV=2	7	1	1	1	136	15.3	11.27	1	1.92
	Q5ZMT0	14-3-3 protein epsilon OS=Gallus gallus OX=9031 GN=YWHAE PE=1 SV=1	5	1	1	1	255	29.2	4.74	1	1.8
	A0A8V0XYP5	Heterogeneous nuclear ribonucleoprotein U OS=Gallus gallus OX=9031 GN=HNRNPU PE=1 SV=1	1	1	1	1	842	91.6	5.94	1	1.71
	**A0A8V1AHR0**	**Keratin OS=Gallus gallus OX=9031 PE=3 SV=1**	**7**	**1**	**1**	**1**	**163**	**17.4**	**4.45**	**1**	**1.65**
	A0A8V0Z1G6	TNF receptor superfamily member 9 OS=Gallus gallus OX=9031 GN=TNFRSF9 PE=4 SV=1	3	1	1	1	373	40	8.88	1	1.64
	F1NN16	40S ribosomal protein S7 OS=Gallus gallus OX=9031 GN=RPS7 PE=1 SV=3	6	1	1	1	194	22.1	10.1	1	1.57
	Q90835	Elongation factor 1-alpha 1 OS=Gallus gallus OX=9031 GN=EEF1A PE=2 SV=1	4	1	1	1	462	50.1	9.01	1	1.49
	P09244	Tubulin beta-7 chain OS=Gallus gallus OX=9031 PE=2 SV=1	2	1	1	1	444	49.6	4.89	1	1.44
	A0A8V0Z557	Heat shock protein HSP 90-alpha OS=Gallus gallus OX=9031 GN=HSP90AA1 PE=3 SV=1	2	1	1	1	734	84.5	5.08	1	1.44
	Q5ZM98	Stress-70 protein. mitochondrial OS=Gallus gallus OX=9031 GN=HSPA9 PE=1 SV=1	2	1	1	1	675	73.1	6.43	1	1.43
	A0A1D5NVR2	Growth hormone receptor OS=Gallus gallus OX=9031 GN=GHR PE=3 SV=1	2	1	1	1	646	72.9	4.69	1	1.36
ID 4158	A0A8V0Z0U1	phosphopyruvate hydratase OS=Gallus gallus OX=9031 GN=ENO1 PE=1 SV=1	6	2	2	2	445	48.7	7.06	2	7.81
	P62801	Histone H4 OS=Gallus gallus OX=9031 GN=H4-VII PE=1 SV=2	29	3	4	3	103	11.4	11.36	3	7.03
	A0A8V1AKG1	Ubiquitin B OS=Gallus gallus OX=9031 GN=LOC101747587 PE=4 SV=1	25	3	3	3	400	44.9	8.75	3	6.73
	P53478	Actin, cytoplasmic type 5 OS=Gallus gallus OX=9031 PE=3 SV=1	3	1	1	1	376	41.8	5.48	1	5.29
	Q5ZMJ6	ADP/ATP translocase OS=Gallus gallus OX=9031 GN=SLC25A4 PE=1 SV=1	8	2	2	2	298	32.9	9.73	2	4.96
	A0A8V0XYP5	Heterogeneous nuclear ribonucleoprotein U OS=Gallus gallus OX=9031 GN=HNRNPU PE=1 SV=1	3	2	2	2	842	91.6	5.94	2	4.65
	A0A8V0ZG96	Pyruvate kinase OS=Gallus gallus OX=9031 GN=PKLR PE=1 SV=1	4	2	2	2	693	75.3	9.25	2	4.39
	K2C1_HUMAN	(Common contaminant protein)	2	1	1	1	643	65.8	8.12	1	4.37
	**Q6PVZ1**	**Keratin, type I cytoskeletal 14 OS=Gallus gallus OX=9031 GN=KRT14 PE=2 SV=1**	**4**	**2**	**2**	**2**	**467**	**51**	**5.07**	**2**	**4.35**
	A0A8V0YK12	ATP synthase subunit alpha OS=Gallus gallus OX=9031 GN=ATP5F1AW PE=1 SV=1	2	1	1	1	619	67.2	8.79	1	3.09
	A0A8V1A5F0	Eukaryotic translation elongation factor 2 OS=Gallus gallus OX=9031 GN=EEF2 PE=1 SV=1	1	1	1	1	881	97.3	6.64	1	3.05
	A0A1D5NVI1	Large ribosomal subunit protein uL6 OS=Gallus gallus OX=9031 GN=RPL9 PE=1 SV=1	14	1	1	1	192	21.8	10.08	1	3
	**A0A3Q2UIQ3**	**Keratin OS=Gallus gallus OX=9031 GN=LOC418813 PE=3 SV=1**	**11**	**1**	**1**	**1**	**98**	**10**	**7.88**	**1**	**2.9**
	Q5ZLN1	Phosphoglycerate mutase 1 OS=Gallus gallus OX=9031 GN=PGAM1 PE=1 SV=3	4	1	1	1	254	28.9	7.49	1	2.9
	A0A8V1A3E8	H1.10 linker histone OS=Gallus gallus OX=9031 GN=LOC121111746 PE=4 SV=1	3	1	1	1	402	41.9	10.62	1	2.84
	A0A8V0YW88	Heterogeneous nuclear ribonucleoprotein K OS=Gallus gallus OX=9031 GN=HNRNPK PE=1 SV=1	2	1	1	1	449	49.7	6.14	1	2.82
	Q5ZME1	Heterousus nuclear ribonucleoprotein A2/B1 OS=Gallus gallus OX=9031 GN=HNRNPA2B1 PE=1 SV=1	3	1	1	1	349	37	8.65	1	2.7

	Accession	Description	Coverage, %	# Peptides	# PSMs	# Unique Peptides	# AAs	MW, kDa	calc. pI	# Peptides *1	|Log Prob| *2
	Q5ZM98	Stress-70 protein. mitochondrial OS=Gallus gallus OX=9031 GN=HSPA9 PE=1 SV=1	2	1	1	1	675	73.1	6.43	1	2.6
	P0CB50	Peroxiredoxin-1 OS=Gallus gallus OX=9031 GN=PRDX1 PE=1 SV=1	5	1	1	1	199	22.3	8.1	1	2.43
	A0A8V0Z4Q7	Large ribosomal subunit protein eL14 OS=Gallus gallus OX=9031 GN=RPL14 PE=1 SV=1	8	1	1	1	155	18.3	10.46	1	2.41
	**A0A8V1AHR0**	**Keratin OS=Gallus gallus OX=9031 PE=3 SV=1**	**7**	**1**	**1**	**1**	**163**	**17.4**	**4.45**	**1**	**2.4**
	A0A8V0Y8F5	Small nuclear ribonucleoprotein Sm D2 OS=Gallus gallus OX=9031 GN=SNRPD2 PE=3 SV=1	6	1	1	1	151	16.9	9.72	1	2.29
	P21566	Cofilin-2 OS=Gallus gallus OX=9031 GN=CFL2 PE=1 SV=2	7	1	1	1	166	18.7	7.88	1	2.28
	A0A8V0X7H3	Small ribosomal subunit protein RACK1 OS=Gallus gallus OX=9031 GN=RACK1 PE=3 SV=1	3	1	1	1	357	39.3	7.65	1	2.16
	**A0A1D5PS82**	**Keratin OS=Gallus gallus OX=9031 GN=LOC121110032 PE=3 SV=1**	**12**	**1**	**1**	**1**	**98**	**10**	**6.24**	**1**	**2.14**
	Q5ZMT0	14-3-3 protein epsilon OS=Gallus gallus OX=9031 GN=YWHAE PE=1 SV=1	5	1	1	1	255	29.2	4.74	1	2.02
	ALBU_BOVIN	(Common contaminant protein)	2	1	1	1	607	69.2	6.18	1	2.01
	P09244	Tubulin beta-7 chain OS=Gallus gallus OX=9031 PE=2 SV=1	2	1	1	1	444	49.6	4.89	1	1.89
	E1BTT8	L-lactate dehydrogenase OS=Gallus gallus OX=9031 GN=LDHA PE=1 SV=2	5	1	1	1	332	36.5	7.9	1	1.81
	**A0A8V1A2T7**	**Keratin 18 OS=Gallus gallus OX=9031 GN=KRT18 PE=1 SV=1**	**2**	**1**	**1**	**1**	**429**	**46.9**	**5.19**	**1**	**1.61**
	K1C9_HUMAN	(Common contaminant protein)	2	1	1	1	623	62.1	5.3	1	1.6
	A0A8V0Z557	Heat shock protein HSP 90-alpha OS=Gallus gallus OX=9031 GN=HSP90AA1 PE=3 SV=1	2	1	1	1	734	84.5	5.08	1	1.4
	P26584	High mobility group protein B2 OS=Gallus gallus OX=9031 GN=HMGB2 PE=2 SV=2	6	1	2	1	207	23.8	8.38	1	0.1
	A0A8V0ZQV3	ITPRIP like 2 OS=Gallus gallus OX=9031 GN=ITPRIPL2 PE=3 SV=1	2	1	1	1	500	54.2	8.82	1	0.1
ID 4257	A0A8V0Z0U1	phosphopyruvate hydratase OS=Gallus gallus OX=9031 GN=ENO1 PE=1 SV=1	6	2	3	2	445	48.7	7.06	2	6.93
	**A0A1D5PS82**	**Keratin OS=Gallus gallus OX=9031 GN=LOC121110032 PE=3 SV=1**	**12**	**2**	**3**	**2**	**98**	**10**	**6.24**	**2**	**6.78**
	Q5ZMJ6	ADP/ATP translocase OS=Gallus gallus OX=9031 GN=SLC25A4 PE=1 SV=1	8	2	2	2	298	32.9	9.73	2	5.63
	P62801	Histone H4 OS=Gallus gallus OX=9031 GN=H4-VII PE=1 SV=2	21	2	2	2	103	11.4	11.36	2	4.11
	P53478	Actin. cytoplasmic type 5 OS=Gallus gallus OX=9031 PE=3 SV=1	4	2	2	2	376	41.8	5.48	2	3.74
	Q5ZLN1	Phosphoglycerate mutase 1 OS=Gallus gallus OX=9031 GN=PGAM1 PE=1 SV=3	4	1	1	1	254	28.9	7.49	1	3.27
	A0A8V0YK12	ATP synthase subunit alpha OS=Gallus gallus OX=9031 GN=ATP5F1AW PE=1 SV=1	2	1	1	1	619	67.2	8.79	1	3.17
	A0A8V1A3F1	Tubulin alpha chain OS=Gallus gallus OX=9031 GN=LOC100859737 PE=3 SV=1	3	1	1	1	435	48.5	5.49	1	3.02
	**A0A8V1A2T7**	**Keratin 18 OS=Gallus gallus OX=9031 GN=KRT18 PE=1 SV=1**	**2**	**1**	**1**	**1**	**429**	**46.9**	**5.19**	**1**	**2.88**
	A0A8V1A3E8	H1.10 linker histone OS=Gallus gallus OX=9031 GN=LOC121111746 PE=4 SV=1	3	1	1	1	402	41.9	10.62	1	2.87
	A0A8V0Z4J0	Ubiquitin carboxyl extension protein 80 OS=Gallus gallus OX=9031 GN=RPS27A PE=3 SV=1	7	1	1	1	233	26.3	10.15	1	2.75
	A0A3Q2UIQ3	Keratin OS=Gallus gallus OX=9031 GN=LOC418813 PE=3 SV=1	12	1	1	1	98	10	7.88	1	2.4
	A0A8V0XYP5	Heterogeneous nuclear ribonucleoprotein U OS=Gallus gallus OX=9031 GN=HNRNPU PE=1 SV=1	2	1	1	1	842	91.6	5.94	1	2.38
	**A0A8V0ZYC5**	**Keratin 75 OS=Gallus gallus OX=9031 GN=KRT75 PE=3 SV=1**	**2**	**1**	**1**	**1**	**536**	**57.5**	**5.96**	**1**	**2.36**
	P50890	Small ribosomal subunit protein uS2 OS=Gallus gallus OX=9031 GN=RPSA PE=3 SV=1	6	1	1	1	296	33	4.87	1	2.35
	P0CB50	Peroxiredoxin-1 OS=Gallus gallus OX=9031 GN=PRDX1 PE=1 SV=1	5	1	1	1	199	22.3	8.1	1	2.33
	A0A8V0Z4Q7	Large ribosomal subunit protein eL14 OS=Gallus gallus OX=9031 GN=RPL14 PE=1 SV=1	8	1	1	1	155	18.3	10.46	1	2.32
	ALBU_BOVIN	(Common contaminant protein)	2	1	1	1	607	69.2	6.18	1	2.23
	E1BTT8	L-lactate dehydrogenase OS=Gallus gallus OX=9031 GN=LDHA PE=1 SV=2	4	1	1	1	332	36.5	7.9	1	2.14
	E1C8Y9	Serine and arginine rich splicing factor 3 OS=Gallus gallus OX=9031 GN=SRSF3 PE=1 SV=1	7	1	1	1	164	19.3	11.65	1	2.1
	**Q6PVZ1**	**Keratin. type I cytoskeletal 14 OS=Gallus gallus OX=9031 GN=KRT14 PE=2 SV=1**	**2**	**1**	**1**	**1**	**467**	**51**	**5.07**	**1**	**1.98**
	A0A8V0ZG96	Pyruvate kinase OS=Gallus gallus OX=9031 GN=PKLR PE=1 SV=1	2	1	1	1	693	75.3	9.25	1	1.98
	A0A8V0X7H3	Small ribosomal subunit protein RACK1 OS=Gallus gallus OX=9031 GN=RACK1 PE=3 SV=1	3	1	1	1	357	39.3	7.65	1	1.74
	Q5ZME1	Heterous nuclear ribonucleoprotein A2/B1 OS=Gallus gallus OX=9031 GN=HNRNPA2B1 PE=1 SV=1	3	1	1	1	349	37	8.65	1	1.69
	Q6ITC7	Small ribosomal subunit protein uS15 OS=Gallus gallus OX=9031 GN=RPS13 PE=2 SV=3	8	1	1	1	151	17.2	10.54	1	1.68
	A0A8V0ZA69	Serine/threonine-protein phosphatase PGAM5, mitochondrial OS=Gallus gallus OX=9031 GN=PGAM5 PE=1 SV=1	3	1	1	1	341	37.2	9.63	1	1.55
	P26584	High mobility group protein B2 OS=Gallus gallus OX=9031 GN=HMGB2 PE=2 SV=2	6	1	1	1	207	23.8	8.38	1	1.53

	Accession	Description	Coverage, %	# Peptides	# PSMs	# Unique Peptides	# AAs	MW, kDa	calc. pI	# Peptides *1	|Log Prob| *2
ID 4259	P53478	Actin, cytoplasmic type 5 OS=Gallus gallus OX=9031 PE=3 SV=1	8	3	4	3	376	41.8	5.48	3	8.3
	P62801	Histone H4 OS=Gallus gallus OX=9031 GN=H4-VII PE=1 SV=2	21	2	2	2	103	11.4	11.36	2	6.69
	P0CB50	Peroxiredoxin-1 OS=Gallus gallus OX=9031 GN=PRDX1 PE=1 SV=1	11	2	2	2	199	22.3	8.1	2	6.46
	P09244	Tubulin beta-7 chain OS=Gallus gallus OX=9031 PE=2 SV=1	6	2	3	2	444	49.6	4.89	2	5.18
	ALBU_BOVIN	(Common contaminant protein)	2	1	1	1	607	69.2	6.18	1	4.21
	Q5ZM98	Stress-70 protein, mitochondrial OS=Gallus gallus OX=9031 GN=HSPA9 PE=1 SV=1	2	1	1	1	675	73.1	6.43	1	3.87
	A0A8V1A3E8	H1.10 linker histone OS=Gallus gallus OX=9031 GN=LOC121111746 PE=4 SV=1	3	1	1	1	402	41.9	10.62	1	3.53
	A0A8V0YK12	ATP synthase subunit alpha OS=Gallus gallus OX=9031 GN=ATP5F1AW PE=1 SV=1	2	1	1	1	619	67.2	8.79	1	3.25
	Q5ZLN1	Phosphoglycerate mutase 1 OS=Gallus gallus OX=9031 GN=PGAM1 PE=1 SV=3	4	1	1	1	254	28.9	7.49	1	3.23
	A0A8V0YV66	Ribosomal protein S4, Y-linked 1 OS=Gallus gallus OX=9031 GN=RPS4Y1 PE=3 SV=1	4	1	1	1	342	37.3	10.42	1	2.82
	Q8JG64	Protein disulfide-isomerase A3 OS=Gallus gallus OX=9031 GN=PDIA3 PE=2 SV=1	2	1	1	1	505	56.1	6.02	1	2.65
	A0A8V0Y9K6	Solute carrier family 25 member 3 OS=Gallus gallus OX=9031 GN=SLC25A3 PE=1 SV=1	3	1	1	1	358	40	9.29	1	2.59
	E1BTT8	L-lactate dehydrogenase OS=Gallus gallus OX=9031 GN=LDHA PE=1 SV=2	4	1	1	1	332	36.5	7.9	1	2.59
	A0A8V0X7H3	Small ribosomal subunit protein RACK1 OS=Gallus gallus OX=9031 GN=RACK1 PE=3 SV=1	3	1	1	1	357	39.3	7.65	1	2.51
	A0A8V1AKG1	Ubiquitin B OS=Gallus gallus OX=9031 GN=LOC101747587 PE=4 SV=1	16	1	1	1	400	44.9	8.75	1	2.49
	Q5ZMJ6	ADP/ATP translocase OS=Gallus gallus OX=9031 GN=SLC25A4 PE=1 SV=1	3	1	1	1	298	32.9	9.73	1	2.46
	A0A8V0Z0U1	phosphopyruvate hydratase OS=Gallus gallus OX=9031 GN=ENO1 PE=1 SV=1	3	1	1	1	445	48.7	7.06	1	2.4
	A0A8V0XYP5	Heterogeneous nuclear ribonucleoprotein U OS=Gallus gallus OX=9031 GN=HNRNPU PE=1 SV=1	2	1	1	1	842	91.6	5.94	1	2.34
	Q9YH06	High mobility group protein B1 OS=Gallus gallus OX=9031 GN=HMGB1 PE=1 SV=1	6	1	1	1	215	24.9	5.74	1	2.13
	**A0A3Q2UIQ3**	**Keratin OS=Gallus gallus OX=9031 GN=LOC418813 PE=3 SV=1**	**12**	**1**	**1**	**1**	**98**	**10**	**7.88**	**1**	**2.08**
	A0A8V1A5F0	Eukaryotic translation elongation factor 2 OS=Gallus gallus OX=9031 GN=EEF2 PE=1 SV=1	1	1	1	1	881	97.3	6.64	1	2.05
	**A0A1D5PS82**	**Keratin OS=Gallus gallus OX=9031 GN=LOC121110032 PE=3 SV=1**	**12**	**1**	**1**	**1**	**98**	**10**	**6.24**	**1**	**1.85**
	Q5F3W3	Mitogen-activated protein kinase 6 OS=Gallus gallus OX=9031 GN=MAPK6 PE=2 SV=1	2	1	1	1	721	82.7	5.07	1	1.77
	A0A1D5NVR2	Growth hormone receptor OS=Gallus gallus OX=9031 GN=GHR PE=3 SV=1	2	1	1	1	646	72.9	4.69	1	1.76
ID 4271	P62801	Histone H4 OS=Gallus gallus OX=9031 GN=H4-VII PE=1 SV=2	21	2	3	2	103	11.4	11.36	2	5.59
	A0A8V0Z0U1	phosphopyruvate hydratase OS=Gallus gallus OX=9031 GN=ENO1 PE=1 SV=1	6	2	2	2	445	48.7	7.06	2	4.35
	A0A8V1A3E8	H1.10 linker histone OS=Gallus gallus OX=9031 GN=LOC121111746 PE=4 SV=1	3	1	1	1	402	41.9	10.62	1	3.47
	Q5ZLN1	Phosphoglycerate mutase 1 OS=Gallus gallus OX=9031 GN=PGAM1 PE=1 SV=3	4	1	1	1	254	28.9	7.49	1	3.46
	A0A8V0Z4Q7	Large ribosomal subunit protein eL14 OS=Gallus gallus OX=9031 GN=RPL14 PE=1 SV=1	8	1	1	1	155	18.3	10.46	1	2.64
	A0A1D5PN54	ATP synthase subunit alpha OS=Gallus gallus OX=9031 GN=ATP5F1AZ PE=1 SV=1	2	1	1	1	553	59.9	9.13	1	2.62
	A0A8V1AKG1	Ubiquitin B OS=Gallus gallus OX=9031 GN=LOC101747587 PE=4 SV=1	16	1	1	1	400	44.9	8.75	1	2.3
	A0A8V0XYP5	Heterogeneous nuclear ribonucleoprotein U OS=Gallus gallus OX=9031 GN=HNRNPU PE=1 SV=1	2	1	1	1	842	91.6	5.94	1	2.29
	A0A8V0X7H3	Small ribosomal subunit protein RACK1 OS=Gallus gallus OX=9031 GN=RACK1 PE=3 SV=1	3	1	1	1	357	39.3	7.65	1	2.26
	Q5ZM98	Stress-70 protein, mitochondrial OS=Gallus gallus OX=9031 GN=HSPA9 PE=1 SV=1	2	1	1	1	675	73.1	6.43	1	2.23
	Q5ZMJ6	ADP/ATP translocase OS=Gallus gallus OX=9031 GN=SLC25A4 PE=1 SV=1	3	1	1	1	298	32.9	9.73	1	2.17
	Q9YH06	High mobility group protein B1 OS=Gallus gallus OX=9031 GN=HMGB1 PE=1 SV=1	6	1	2	1	215	24.9	5.74	1	2.15
	P0CB50	Peroxiredoxin-1 OS=Gallus gallus OX=9031 GN=PRDX1 PE=1 SV=1	5	1	1	1	199	22.3	8.1	1	1.95
	Q5ZME1	Heterootus nuclear ribonucleoprotein A2/B1 OS=Gallus gallus OX=9031 GN=HNRNPA2B1 PE=1 SV=1	3	1	1	1	349	37	8.65	1	1.91
	A0A8V0Z557	Heat shock protein HSP 90-alpha OS=Gallus gallus OX=9031 GN=HSP90AA1 PE=3 SV=1	2	1	1	1	734	84.5	5.08	1	1.6
	A0A8V0ZQV3	ITPRIP like 2 OS=Gallus gallus OX=9031 GN=ITPRIPL2 PE=3 SV=1	2	1	1	1	500	54.2	8.82	1	0.1

ID 4293	Accession	Description	Coverage, %	# Peptides	# PSMs	# Unique Peptides	# AAs	MW, kDa	calc. pI	# Peptides *1	|Log Prob| *2
	P0CB50	Peroxiredoxin-1 OS=Gallus gallus OX=9031 GN=PRDX1 PE=1 SV=1	11	2	2	2	199	22.3	8.1	2	11
	A0A8V1AKG1	Ubiquitin B OS=Gallus gallus OX=9031 GN=LOC101747587 PE=4 SV=1	25	2	2	2	400	44.9	8.75	2	25
	A0A8V0Z0U1	phosphopyruvate hydratase OS=Gallus gallus OX=9031 GN=ENO1 PE=1 SV=1	6	2	2	2	445	48.7	7.06	2	6
	P62801	Histone H4 OS=Gallus gallus OX=9031 GN=H4-VII PE=1 SV=2	21	2	2	2	103	11.4	11.36	2	21
	A0A8V0YK12	ATP synthase subunit alpha OS=Gallus gallus OX=9031 GN=ATP5F1AW PE=1 SV=1	5	2	2	2	619	67.2	8.79	2	5
	Q5ZIQ3	Heterogeneous nuclear ribonucleoprotein K OS=Gallus gallus OX=9031 GN=HNRNPK PE=2 SV=1	2	1	1	1	427	47.2	6.47	1	2
	Q5ZLN1	Phosphoglycerate mutase 1 OS=Gallus gallus OX=9031 GN=PGAM1 PE=1 SV=3	4	1	1	1	254	28.9	7.49	1	4
	A0A8V1A3E8	H1.10 linker histone OS=Gallus gallus OX=9031 GN=LOC121111746 PE=4 SV=1	3	1	1	1	402	41.9	10.62	1	3
	A0A8V0X7H3	Small ribosomal subunit protein RACK1 OS=Gallus gallus OX=9031 GN=RACK1 PE=3 SV=1	3	1	1	1	357	39.3	7.65	1	3
	P26584	High mobility group protein B2 OS=Gallus gallus OX=9031 GN=HMGB2 PE=2 SV=2	6	1	4	1	207	23.8	8.38	1	6
	Q6ITC7	Small ribosomal subunit protein uS15 OS=Gallus gallus OX=9031 GN=RPS13 PE=2 SV=3	8	1	1	1	151	17.2	10.54	1	8
	A0A8V0ZG96	Pyruvate kinase OS=Gallus gallus OX=9031 GN=PKLR PE=1 SV=1	2	1	1	1	693	75.3	9.25	1	2
	A0A8V0Z4Q7	Large ribosomal subunit protein eL14 OS=Gallus gallus OX=9031 GN=RPL14 PE=1 SV=1	8	1	1	1	155	18.3	10.46	1	8
	ALBU_BOVIN	(Common contaminant protein)	2	1	1	1	607	69.2	6.18	1	2
	**Q6PVZ1**	**Keratin. type I cytoskeletal 14 OS=Gallus gallus OX=9031 GN=KRT14 PE=2 SV=1**	**2**	**1**	**1**	**1**	**467**	**51**	**5.07**	**1**	**2**
	P53478	Actin, cytoplasmic type 5 OS=Gallus gallus OX=9031 PE=3 SV=1	3	1	1	1	376	41.8	5.48	1	3
	A0A8V0Y5D3	Nucleophosmin OS=Gallus gallus OX=9031 GN=NPM1 PE=1 SV=1	7	1	1	1	307	34.1	4.83	1	7
	Q5ZM98	Stress-70 protein, mitochondrial OS=Gallus gallus OX=9031 GN=HSPA9 PE=1 SV=1	2	1	1	1	675	73.1	6.43	1	2
	A0A8V0XYP5	Heterogeneous nuclear ribonucleoprotein U OS=Gallus gallus OX=9031 GN=HNRNPU PE=1 SV=1	2	1	1	1	842	91.6	5.94	1	2
	P09244	Tubulin beta-7 chain OS=Gallus gallus OX=9031 PE=2 SV=1	2	1	1	1	444	49.6	4.89	1	2
	A0A8V0Z557	Heat shock protein HSP 90-alpha OS=Gallus gallus OX=9031 GN=HSP90AA1 PE=3 SV=1	2	1	1	1	734	84.5	5.08	1	2
	R4GIS3	Nuclear receptor interacting protein 1 OS=Gallus gallus OX=9031 GN=NRIP1 PE=4 SV=3	1	1	1	1	1155	126.8	8.46	1	1
ID 4294	P62801	Histone H4 OS=Gallus gallus OX=9031 GN=H4-VII PE=1 SV=2	21	2	4	2	103	11.4	11.36	2	5.89
	P09244	Tubulin beta-7 chain OS=Gallus gallus OX=9031 PE=2 SV=1	5	2	2	2	444	49.6	4.89	2	4.4
	P53478	Actin. cytoplasmic type 5 OS=Gallus gallus OX=9031 PE=3 SV=1	7	2	2	2	376	41.8	5.48	2	4.08
	Q5ZMJ6	ADP/ATP translocase OS=Gallus gallus OX=9031 GN=SLC25A4 PE=1 SV=1	8	2	2	2	298	32.9	9.73	2	3.76
	ALBU_BOVIN	(Common contaminant protein)	3	2	2	2	607	69.2	6.18	2	3.76
	A0A8V1A3E8	H1.10 linker histone OS=Gallus gallus OX=9031 GN=LOC121111746 PE=4 SV=1	3	1	1	1	402	41.9	10.62	1	3.54
	A0A8V0Z0U1	phosphopyruvate hydratase OS=Gallus gallus OX=9031 GN=ENO1 PE=1 SV=1	3	1	1	1	445	48.7	7.06	1	3.13
	P0CB50	Peroxiredoxin-1 OS=Gallus gallus OX=9031 GN=PRDX1 PE=1 SV=1	5	1	1	1	199	22.3	8.1	1	2.85
	A0A8V0Z4Q7	Large ribosomal subunit protein eL14 OS=Gallus gallus OX=9031 GN=RPL14 PE=1 SV=1	8	1	1	1	155	18.3	10.46	1	2.75
	A0A8V0YK12	ATP synthase subunit alpha OS=Gallus gallus OX=9031 GN=ATP5F1AW PE=1 SV=1	2	1	1	1	619	67.2	8.79	1	2.75
	Q5ZLN1	Phosphoglycerate mutase 1 OS=Gallus gallus OX=9031 GN=PGAM1 PE=1 SV=3	4	1	1	1	254	28.9	7.49	1	2.22
	A0A8V0ZG96	Pyruvate kinase OS=Gallus gallus OX=9031 GN=PKLR PE=1 SV=1	2	1	1	1	693	75.3	9.25	1	2.14
	E1BTT8	L-lactate dehydrogenase OS=Gallus gallus OX=9031 GN=LDHA PE=1 SV=2	4	1	1	1	332	36.5	7.9	1	2.08
	A0A8V1AKG1	Ubiquitin B OS=Gallus gallus OX=9031 GN=LOC101747587 PE=4 SV=1	16	1	1	1	400	44.9	8.75	1	1.82
	Q04619	Heat shock cognate protein HSP 90-beta OS=Gallus gallus OX=9031 GN=HSP90AB1 PE=2 SV=1	2	1	1	1	725	83.4	5.02	1	1.74
	Q5ZKC9	14-3-3 protein zeta OS=Gallus gallus OX=9031 GN=YWHAZ PE=2 SV=1	4	1	1	1	245	27.8	4.79	1	1.73
	**A0A1D5PS82**	**Keratin OS=Gallus gallus OX=9031 GN=LOC121110032 PE=3 SV=1**	**12**	**1**	**1**	**1**	**98**	**10**	**6.24**	**1**	**1.73**
	**A0A8V0ZYC5**	**Keratin 75 OS=Gallus gallus OX=9031 GN=KRT75 PE=3 SV=1**	**2**	**1**	**1**	**1**	**536**	**57.5**	**5.96**	**1**	**1.71**
	A0A8V0XYP5	Heterogeneous nuclear ribonucleoprotein U OS=Gallus gallus OX=9031 GN=HNRNPU PE=1 SV=1	2	1	1	1	842	91.6	5.94	1	1.7
	Q90835	Elongation factor 1-alpha 1 OS=Gallus gallus OX=9031 GN=EEF1A PE=2 SV=1	4	1	1	1	462	50.1	9.01	1	1.63
	A0A1D5NVR2	Growth hormone receptor OS=Gallus gallus OX=9031 GN=GHR PE=3 SV=1	2	1	1	1	646	72.9	4.69	1	1.47
	A0A8V0ZA69	Serine/threonine-protein phosphatase PGAM5. mitochondrial OS=Gallus gallus OX=9031 GN=PGAM5 PE=1 SV=1	3	1	1	1	341	37.2	9.63	1	1.32
	P26584	High mobility group protein B2 OS=Gallus gallus OX=9031 GN=HMGB2 PE=2 SV=2	6	1	2	1	207	23.8	8.38	1	0.1

ID 4499	Accession	Description	Coverage, %	# Peptides	# PSMs	# Unique Peptides	# AAs	MW, kDa	calc. pI	# Peptides *1	|Log Prob| *2
	**P20308_CFP**	**Feather keratin 4 OS=Gallus gallus OX=9031 PE=3 SV=2 [CFP_A]**	**60**	**26**	**52**	**26**	**98**	**10.1**	**7.62**	**26**	**41.44**
	A0A8V1AKG1	Ubiquitin B OS=Gallus gallus OX=9031 GN=LOC101747587 PE=4 SV=1	25	3	4	3	400	44.9	8.75	3	5.98
	P62801	Histone H4 OS=Gallus gallus OX=9031 GN=H4-VII PE=1 SV=2	29	3	4	3	103	11.4	11.36	3	5.78
	A0A8V0Z0U1	phosphopyruvate hydratase OS=Gallus gallus OX=9031 GN=ENO1 PE=1 SV=1	12	4	4	4	445	48.7	7.06	4	5.52
	A0A8V1A5Y2	Heat shock 70kDa protein 8 OS=Gallus gallus OX=9031 GN=HSPA8 PE=1 SV=1	10	4	4	4	705	78	5.85	4	5.22
	A0A8V0ZG96	Pyruvate kinase OS=Gallus gallus OX=9031 GN=PKLR PE=1 SV=1	4	2	3	2	693	75.3	9.25	2	4.65
	P09244	Tubulin beta-7 chain OS=Gallus gallus OX=9031 PE=2 SV=1	9	3	5	3	444	49.6	4.89	3	4.42
	**A0A1D5P6V7**	**Keratin OS=Gallus gallus OX=9031 GN=LOC121113098 PE=3 SV=1**	**16**	**3**	**7**	**3**	**98**	**9.9**	**7.52**	**3**	**4.15**
	A0A8V0YK12	ATP synthase subunit alpha OS=Gallus gallus OX=9031 GN=ATP5F1AW PE=1 SV=1	6	3	3	3	619	67.2	8.79	3	3.8
	A0A8V1A3E8	H1.10 linker histone OS=Gallus gallus OX=9031 GN=LOC121111746 PE=4 SV=1	3	1	1	1	402	41.9	10.62	1	3.59
	A0A8V1A624	ATP synthase subunit beta OS=Gallus gallus OX=9031 GN=ATP5B PE=1 SV=1	4	1	1	1	533	56.6	5.66	1	3.49
	A0A8V0XYP5	Heterogeneous nuclear ribonucleoprotein U OS=Gallus gallus OX=9031 GN=HNRNPU PE=1 SV=1	3	2	2	2	842	91.6	5.94	2	3.44
	Q5ZLN1	Phosphoglycerate mutase 1 OS=Gallus gallus OX=9031 GN=PGAM1 PE=1 SV=3	4	1	1	1	254	28.9	7.49	1	3.35
	**A0A8V1AH33**	**Keratin OS=Gallus gallus OX=9031 GN=LOC121107567 PE=3 SV=1**	**14**	**1**	**1**	**1**	**98**	**9.8**	**7.56**	**1**	**3.32**
	P0CB50	Peroxiredoxin-1 OS=Gallus gallus OX=9031 GN=PRDX1 PE=1 SV=1	11	2	2	2	199	22.3	8.1	2	3.23
	Q5ZMJ6	ADP/ATP translocase OS=Gallus gallus OX=9031 GN=SLC25A4 PE=1 SV=1	8	2	2	2	298	32.9	9.73	2	3.14
	**A0A8V0ZGG3**	**Keratin OS=Gallus gallus OX=9031 GN=LOC112533192 PE=3 SV=1**	**12**	**2**	**3**	**2**	**129**	**13.2**	**8.12**	**2**	**3.1**
	P53478	Actin. cytoplasmic type 5 OS=Gallus gallus OX=9031 PE=3 SV=1	7	3	3	3	376	41.8	5.48	3	2.87
	P50890	Small ribosomal subunit protein uS2 OS=Gallus gallus OX=9031 GN=RPSA PE=3 SV=1	9	2	2	2	296	33	4.87	2	2.65
	A0A8V0X7H3	Small ribosomal subunit protein RACK1 OS=Gallus gallus OX=9031 GN=RACK1 PE=3 SV=1	3	1	1	1	357	39.3	7.65	1	2.64
	A0A8V0Z4Q7	Large ribosomal subunit protein eL14 OS=Gallus gallus OX=9031 GN=RPL14 PE=1 SV=1	8	1	1	1	155	18.3	10.46	1	2.6
	Q5ZME1	Heteroous nuclear ribonucleoprotein A2/B1 OS=Gallus gallus OX=9031 GN=HNRNPA2B1 PE=1 SV=1	3	1	1	1	349	37	8.65	1	2.53
	**A0A8V1AL30**	**Keratin OS=Gallus gallus OX=9031 GN=LOC100859191 PE=3 SV=1**	**12**	**1**	**4**	**1**	**98**	**9.7**	**7.52**	**1**	**2.44**
	Q5ZM98	Stress-70 protein. mitochondrial OS=Gallus gallus OX=9031 GN=HSPA9 PE=1 SV=1	2	1	1	1	675	73.1	6.43	1	2.34
	A0A8V0YV66	Ribosomal protein S4. Y-linked 1 OS=Gallus gallus OX=9031 GN=RPS4Y1 PE=3 SV=1	4	1	2	1	342	37.3	10.42	1	1.95
ID 4505	A0A8V0Z0U1	phosphopyruvate hydratase OS=Gallus gallus OX=9031 GN=ENO1 PE=1 SV=1	8	3	4	3	445	48.7	7.06	3	8.43
	**A0A8V1AGY1**	**Keratin OS=Gallus gallus OX=9031 GN=LOC426913 PE=3 SV=1**	**11**	**2**	**5**	**2**	**131**	**13.7**	**7.09**	**2**	**7.01**
	P62801	Histone H4 OS=Gallus gallus OX=9031 GN=H4-VII PE=1 SV=2	21	2	2	2	103	11.4	11.36	2	6.47
	ALBU_BOVIN	(Common contaminant protein)	3	2	2	2	607	69.2	6.18	2	5.99
	P53478	Actin. cytoplasmic type 5 OS=Gallus gallus OX=9031 PE=3 SV=1	5	2	2	2	376	41.8	5.48	2	5.96
	A0A8V1A3F1	Tubulin alpha chain OS=Gallus gallus OX=9031 GN=LOC100859737 PE=3 SV=1	7	2	2	2	435	48.5	5.49	2	5.77
	Q5ZMJ6	ADP/ATP translocase OS=Gallus gallus OX=9031 GN=SLC25A4 PE=1 SV=1	8	2	2	2	298	32.9	9.73	2	5.52
	A0A8V1AKG1	Ubiquitin B OS=Gallus gallus OX=9031 GN=LOC101747587 PE=4 SV=1	25	2	2	2	400	44.9	8.75	2	4.98
	Q90835	Elongation factor 1-alpha 1 OS=Gallus gallus OX=9031 GN=EEF1A PE=2 SV=1	6	2	2	2	462	50.1	9.01	2	4.32
	A0A8V0YK12	ATP synthase subunit alpha OS=Gallus gallus OX=9031 GN=ATP5F1AW PE=1 SV=1	2	1	1	1	619	67.2	8.79	1	3.77
	A0A1D5NVI1	Large ribosomal subunit protein uL6 OS=Gallus gallus OX=9031 GN=RPL9 PE=1 SV=1	14	1	1	1	192	21.8	10.08	1	3.73
	E1BTT8	L-lactate dehydrogenase OS=Gallus gallus OX=9031 GN=LDHA PE=1 SV=2	4	1	1	1	332	36.5	7.9	1	3.53
	A0A8V0YV66	Ribosomal protein S4. Y-linked 1 OS=Gallus gallus OX=9031 GN=RPS4Y1 PE=3 SV=1	4	1	1	1	342	37.3	10.42	1	3.25
	A0A1L1RUW7	Deoxyuridine 5'-triphosphate nucleotidohydrolase OS=Gallus gallus OX=9031 GN=DUT PE=1 SV=2	6	1	1	1	187	20.2	8.88	1	3.08
	A0A8V0XYP5	Heterogeneous nuclear ribonucleoprotein U OS=Gallus gallus OX=9031 GN=HNRNPU PE=1 SV=1	2	1	1	1	842	91.6	5.94	1	3.01
	A0A8V1A3E8	H1.10 linker histone OS=Gallus gallus OX=9031 GN=LOC121111746 PE=4 SV=1	3	1	1	1	402	41.9	10.62	1	3
	A0A8V0ZFP5	Phosphoglycerate mutase OS=Gallus gallus OX=9031 GN=PGAM1 PE=3 SV=1	3	1	1	1	331	37.3	6.86	1	2.92
	Q5ZIQ3	Heterogeneous nuclear ribonucleoprotein K OS=Gallus gallus OX=9031 GN=HNRNPK PE=2 SV=1	5	1	1	1	427	47.2	6.47	1	2.9
	A0A8V0ZG96	Pyruvate kinase OS=Gallus gallus OX=9031 GN=PKLR PE=1 SV=1	2	1	1	1	693	75.3	9.25	1	2.9
	P0CB50	Peroxiredoxin-1 OS=Gallus gallus OX=9031 GN=PRDX1 PE=1 SV=1	5	1	1	1	199	22.3	8.1	1	2.7

	Accession	Description	Coverage, %	# Peptides	# PSMs	# Unique Peptides	# AAs	MW, kDa	calc. pI	# Peptides *1	|Log Prob| *2
	A0A8V0X7H3	Small ribosomal subunit protein RACK1 OS=Gallus gallus OX=9031 GN=RACK1 PE=3 SV=1	3	1	1	1	357	39.3	7.65	1	2.7
	Q5ZM98	Stress-70 protein, mitochondrial OS=Gallus gallus OX=9031 GN=HSPA9 PE=1 SV=1	2	1	1	1	675	73.1	6.43	1	2.65
	Q9W719	Hypoxanthine-guanine phosphoribosyltransferase OS=Gallus gallus OX=9031 GN=HPRT1 PE=2 SV=1	6	1	1	1	218	24.6	7.42	1	2.54
	A0A8V0ZA69	Serine/threonine-protein phosphatase PGAM5. mitochondrial OS=Gallus gallus OX=9031 GN=PGAM5 PE=1 SV=1	3	1	1	1	341	37.2	9.63	1	2.48
	A0A8V1A8R2	40S ribosomal protein S8 OS=Gallus gallus OX=9031 GN=RPS8 PE=1 SV=1	6	1	1	1	267	30.7	10.42	1	2.46
	Q9DEA3	Proliferating cell nuclear antigen OS=Gallus gallus OX=9031 GN=PCNA PE=1 SV=1	7	1	1	1	262	28.9	4.72	1	2.4
	A0A8V0Z4Q7	Large ribosomal subunit protein eL14 OS=Gallus gallus OX=9031 GN=RPL14 PE=1 SV=1	8	1	1	1	155	18.3	10.46	1	2.39
	P47826	Large ribosomal subunit protein uL10 OS=Gallus gallus OX=9031 GN=RPLP0 PE=2 SV=1	3	1	1	1	316	34.3	5.99	1	2.36
	Q9YH06	High mobility group protein B1 OS=Gallus gallus OX=9031 GN=HMGB1 PE=1 SV=1	6	1	2	1	215	24.9	5.74	1	2.34
	Q5ZMT0	14-3-3 protein epsilon OS=Gallus gallus OX=9031 GN=YWHAE PE=1 SV=1	5	1	1	1	255	29.2	4.74	1	2.18
	Q5ZME1	Heteroous nuclear ribonucleoprotein A2/B1 OS=Gallus gallus OX=9031 GN=HNRNPA2B1 PE=1 SV=1	3	1	1	1	349	37	8.65	1	2.01
	P50890	Small ribosomal subunit protein uS2 OS=Gallus gallus OX=9031 GN=RPSA PE=3 SV=1	6	1	1	1	296	33	4.87	1	1.99
	A0A8V0Z557	Heat shock protein HSP 90-alpha OS=Gallus gallus OX=9031 GN=HSP90AA1 PE=3 SV=1	1	1	1	1	734	84.5	5.08	1	1.89
	A0A8V0ZSX1	Myosin IE OS=Gallus gallus OX=9031 GN=MYO1E PE=1 SV=1	1	1	1	1	1176	134.2	9.04	1	1.54
ID 4513	**A0A8V1AH20**	**Keratin OS=Gallus gallus OX=9031 GN=LOC100859427 PE=3 SV=1**	**22**	**4**	**5**	**4**	**98**	**9.9**	**7.94**	**4**	**16.18**
	A0A8V1AKG1	Ubiquitin B OS=Gallus gallus OX=9031 GN=LOC101747587 PE=4 SV=1	25	3	4	3	400	44.9	8.75	3	12.14
	A0A8V0Z0U1	phosphopyruvate hydratase OS=Gallus gallus OX=9031 GN=ENO1 PE=1 SV=1	9	3	3	3	445	48.7	7.06	3	11.28
	P62801	Histone H4 OS=Gallus gallus OX=9031 GN=H4-VII PE=1 SV=2	21	2	2	2	103	11.4	11.36	2	7.75
	P53478	Actin, cytoplasmic type 5 OS=Gallus gallus OX=9031 PE=3 SV=1	7	3	3	3	376	41.8	5.48	3	7.34
	A0A8V0YK12	ATP synthase subunit alpha OS=Gallus gallus OX=9031 GN=ATP5F1AW PE=1 SV=1	5	2	2	2	619	67.2	8.79	2	5.83
	Q5ZMJ6	ADP/ATP translocase OS=Gallus gallus OX=9031 GN=SLC25A4 PE=1 SV=1	8	2	2	2	298	32.9	9.73	2	5.53
	Q90835	Elongation factor 1-alpha 1 OS=Gallus gallus OX=9031 GN=EEF1A PE=2 SV=1	6	2	2	2	462	50.1	9.01	2	4.89
	A0A8V0ZG96	Pyruvate kinase OS=Gallus gallus OX=9031 GN=PKLR PE=1 SV=1	2	1	1	1	693	75.3	9.25	1	4.63
	P09244	Tubulin beta-7 chain OS=Gallus gallus OX=9031 PE=2 SV=1	2	1	1	1	444	49.6	4.89	1	4.34
	A0A8V1A3E8	H1.10 linker histone OS=Gallus gallus OX=9031 GN=LOC121111746 PE=4 SV=1	3	1	1	1	402	41.9	10.62	1	4.06
	A0A8V0XYP5	Heterogeneous nuclear ribonucleoprotein U OS=Gallus gallus OX=9031 GN=HNRNPU PE=1 SV=1	2	1	1	1	842	91.6	5.94	1	3.79
	Q5ZLN1	Phosphoglycerate mutase 1 OS=Gallus gallus OX=9031 GN=PGAM1 PE=1 SV=3	4	1	1	1	254	28.9	7.49	1	3.69
	A0A8V1A3F1	Tubulin alpha chain OS=Gallus gallus OX=9031 GN=LOC100859737 PE=3 SV=1	3	1	1	1	435	48.5	5.49	1	3.36
	A0A8V0Z4Q7	Large ribosomal subunit protein eL14 OS=Gallus gallus OX=9031 GN=RPL14 PE=1 SV=1	8	1	1	1	155	18.3	10.46	1	3.32
	**A0A1D5PS82**	**Keratin OS=Gallus gallus OX=9031 GN=LOC121110032 PE=3 SV=1**	**12**	**1**	**3**	**1**	**98**	**10**	**6.24**	**1**	**3.24**
	A0A8V1A8R2	40S ribosomal protein S8 OS=Gallus gallus OX=9031 GN=RPS8 PE=1 SV=1	6	1	1	1	267	30.7	10.42	1	3.16
	Q5ZM98	Stress-70 protein, mitochondrial OS=Gallus gallus OX=9031 GN=HSPA9 PE=1 SV=1	2	1	1	1	675	73.1	6.43	1	3.15
	P0CB50	Peroxiredoxin-1 OS=Gallus gallus OX=9031 GN=PRDX1 PE=1 SV=1	5	1	1	1	199	22.3	8.1	1	3.1
	CASK_BOVIN	(Common contaminant protein)	5	1	1	1	190	21.3	6.77	1	3.06
	A0A8V0X7H3	Small ribosomal subunit protein RACK1 OS=Gallus gallus OX=9031 GN=RACK1 PE=3 SV=1	3	1	1	1	357	39.3	7.65	1	3.03
	A0A8V0YV66	Ribosomal protein S4, Y-linked 1 OS=Gallus gallus OX=9031 GN=RPS4Y1 PE=3 SV=1	4	1	1	1	342	37.3	10.42	1	2.9
	A0A1D5PSE5	ATP-citrate synthase OS=Gallus gallus OX=9031 GN=ACLY PE=1 SV=1	2	1	1	1	1101	121.1	7.72	1	2.86
	A0A8V0Z0N7	Peptidyl-prolyl cis-trans isomerase OS=Gallus gallus OX=9031 GN=PPIA PE=1 SV=1	9	1	1	1	154	16.6	8.35	1	2.75
	**A0A8V1A2T7**	**Keratin 18 OS=Gallus gallus OX=9031 GN=KRT18 PE=1 SV=1**	**2**	**1**	**1**	**1**	**429**	**46.9**	**5.19**	**1**	**2.65**
	A0A8V1A5Y2	Heat shock 70kDa protein 8 OS=Gallus gallus OX=9031 GN=HSPA8 PE=1 SV=1	2	1	1	1	705	78	5.85	1	2.27
	Q9YH06	High mobility group protein B1 OS=Gallus gallus OX=9031 GN=HMGB1 PE=1 SV=1	6	1	3	1	215	24.9	5.74	1	2.01
	Q5ZMT0	14-3-3 protein epsilon OS=Gallus gallus OX=9031 GN=YWHAE PE=1 SV=1	5	1	1	1	255	29.2	4.74	1	1.98

ID	Accession	Description	Coverage, %	# Peptides	# PSMs	# Unique Peptides	# AAs	MW, kDa	calc. pI	# Peptides *1	|Log Prob| *2
ID 4552	P62801	Histone H4 OS=Gallus gallus OX=9031 GN=H4-VII PE=1 SV=2	21	2	3	2	103	11.4	11.36	2	4.43
	A0A8V0Z0U1	phosphopyruvate hydratase OS=Gallus gallus OX=9031 GN=ENO1 PE=1 SV=1	3	1	1	1	445	48.7	7.06	1	4.36
	A0A8V1AKG1	Ubiquitin B OS=Gallus gallus OX=9031 GN=LOC101747587 PE=4 SV=1	25	2	2	2	400	44.9	8.75	2	3.89
	Q5ZMJ6	ADP/ATP translocase OS=Gallus gallus OX=9031 GN=SLC25A4 PE=1 SV=1	4	1	1	1	298	32.9	9.73	1	3.76
	Q5ZMT0	14-3-3 protein epsilon OS=Gallus gallus OX=9031 GN=YWHAE PE=1 SV=1	9	2	2	2	255	29.2	4.74	2	3.47
	A0A8V1A3E8	H1.10 linker histone OS=Gallus gallus OX=9031 GN=LOC121111746 PE=4 SV=1	3	1	1	1	402	41.9	10.62	1	3.33
	A0A8V0XYP5	Heterogeneous nuclear ribonucleoprotein U OS=Gallus gallus OX=9031 GN=HNRNPU PE=1 SV=1	2	1	1	1	842	91.6	5.94	1	2.54
	A0A8V0YK12	ATP synthase subunit alpha OS=Gallus gallus OX=9031 GN=ATP5F1AW PE=1 SV=1	2	1	1	1	619	67.2	8.79	1	2.35
	P53478	Actin, cytoplasmic type 5 OS=Gallus gallus OX=9031 PE=3 SV=1	3	1	1	1	376	41.8	5.48	1	2.14
	A0A8V1A5F0	Eukaryotic translation elongation factor 2 OS=Gallus gallus OX=9031 GN=EEF2 PE=1 SV=1	1	1	1	1	881	97.3	6.64	1	2.12
	P0CB50	Peroxiredoxin-1 OS=Gallus gallus OX=9031 GN=PRDX1 PE=1 SV=1	5	1	1	1	199	22.3	8.1	1	2.1
	Q5ZLN1	Phosphoglycerate mutase 1 OS=Gallus gallus OX=9031 GN=PGAM1 PE=1 SV=3	4	1	1	1	254	28.9	7.49	1	2.08
	A0A8V0ZA69	Serine/threonine-protein phosphatase PGAM5. mitochondrial OS=Gallus gallus OX=9031 GN=PGAM5 PE=1 SV=1	3	1	1	1	341	37.2	9.63	1	2.01
	A0A8V0Z4Q7	Large ribosomal subunit protein eL14 OS=Gallus gallus OX=9031 GN=RPL14 PE=1 SV=1	8	1	1	1	155	18.3	10.46	1	2
	A0A8V0X7H3	Small ribosomal subunit protein RACK1 OS=Gallus gallus OX=9031 GN=RACK1 PE=3 SV=1	3	1	1	1	357	39.3	7.65	1	1.92
	**A0A8V0ZYC5**	**Keratin 75 OS=Gallus gallus OX=9031 GN=KRT75 PE=3 SV=1**	**2**	**1**	**1**	**1**	**536**	**57.5**	**5.96**	**1**	**1.84**
	A0A8V1A3F1	Tubulin alpha chain OS=Gallus gallus OX=9031 GN=LOC100859737 PE=3 SV=1	3	1	1	1	435	48.5	5.49	1	1.84
	**A0A8V1A2T7**	**Keratin 18 OS=Gallus gallus OX=9031 GN=KRT18 PE=1 SV=1**	**2**	**1**	**1**	**1**	**429**	**46.9**	**5.19**	**1**	**1.79**
ID 4571	P62801	Histone H4 OS=Gallus gallus OX=9031 GN=H4-VII PE=1 SV=2	21	2	2	2	103	11.4	11.36	2	5.36
	A0A8V1AKG1	Ubiquitin B OS=Gallus gallus OX=9031 GN=LOC101747587 PE=4 SV=1	25	2	2	2	400	44.9	8.75	2	4.5
	P53478	Actin, cytoplasmic type 5 OS=Gallus gallus OX=9031 PE=3 SV=1	5	2	2	2	376	41.8	5.48	2	4.45
	Q5ZME1	Heterous nuclear ribonucleoprotein A2/B1 OS=Gallus gallus OX=9031 GN=HNRNPA2B1 PE=1 SV=1	7	2	2	2	349	37	8.65	2	4.45
	A0A8V0Z0U1	phosphopyruvate hydratase OS=Gallus gallus OX=9031 GN=ENO1 PE=1 SV=1	6	2	2	2	445	48.7	7.06	2	3.7
	A0A8V0YK12	ATP synthase subunit alpha OS=Gallus gallus OX=9031 GN=ATP5F1AW PE=1 SV=1	2	1	1	1	619	67.2	8.79	1	3.44
	**Q6PVZ1**	**Keratin. type I cytoskeletal 14 OS=Gallus gallus OX=9031 GN=KRT14 PE=2 SV=1**	**4**	**2**	**2**	**2**	**467**	**51**	**5.07**	**2**	**3.42**
	Q5ZLN1	Phosphoglycerate mutase 1 OS=Gallus gallus OX=9031 GN=PGAM1 PE=1 SV=3	4	1	1	1	254	28.9	7.49	1	3.3
	**A0A8V1A2T7**	**Keratin 18 OS=Gallus gallus OX=9031 GN=KRT18 PE=1 SV=1**	**2**	**1**	**1**	**1**	**429**	**46.9**	**5.19**	**1**	**2.92**
	A0A8V1A3E8	H1.10 linker histone OS=Gallus gallus OX=9031 GN=LOC121111746 PE=4 SV=1	3	1	1	1	402	41.9	10.62	1	2.83
	A0A8V1A3F1	Tubulin alpha chain OS=Gallus gallus OX=9031 GN=LOC100859737 PE=3 SV=1	3	1	1	1	435	48.5	5.49	1	2.81
	A0A8V0ZG96	Pyruvate kinase OS=Gallus gallus OX=9031 GN=PKLR PE=1 SV=1	2	1	1	1	693	75.3	9.25	1	2.66
	A0A8V0Z4Q7	Large ribosomal subunit protein eL14 OS=Gallus gallus OX=9031 GN=RPL14 PE=1 SV=1	8	1	1	1	155	18.3	10.46	1	2.61
	Q9YH06	High mobility group protein B1 OS=Gallus gallus OX=9031 GN=HMGB1 PE=1 SV=1	6	1	3	1	215	24.9	5.74	1	2.48
	A0A8V0YV66	Ribosomal protein S4. Y-linked 1 OS=Gallus gallus OX=9031 GN=RPS4Y1 PE=3 SV=1	4	1	1	1	342	37.3	10.42	1	2.43
	A0A8V0XYP5	Heterogeneous nuclear ribonucleoprotein U OS=Gallus gallus OX=9031 GN=HNRNPU PE=1 SV=1	2	1	1	1	842	91.6	5.94	1	2.25
	P0CB50	Peroxiredoxin-1 OS=Gallus gallus OX=9031 GN=PRDX1 PE=1 SV=1	5	1	1	1	199	22.3	8.1	1	2.22
	**A0A8V0ZYC5**	**Keratin 75 OS=Gallus gallus OX=9031 GN=KRT75 PE=3 SV=1**	**2**	**1**	**1**	**1**	**536**	**57.5**	**5.96**	**1**	**1.95**
	E1BTT8	L-lactate dehydrogenase OS=Gallus gallus OX=9031 GN=LDHA PE=1 SV=2	4	1	1	1	332	36.5	7.9	1	1.93
	P09244	Tubulin beta-7 chain OS=Gallus gallus OX=9031 PE=2 SV=1	3	1	1	1	444	49.6	4.89	1	1.81
	Q5ZMJ6	ADP/ATP translocase OS=Gallus gallus OX=9031 GN=SLC25A4 PE=1 SV=1	3	1	1	1	298	32.9	9.73	1	1.79
	A0A8V0Y5D3	Nucleophosmin OS=Gallus gallus OX=9031 GN=NPM1 PE=1 SV=1	7	1	1	1	307	34.1	4.83	1	1.78
	P50890	Small ribosomal subunit protein uS2 OS=Gallus gallus OX=9031 GN=RPSA PE=3 SV=1	6	1	1	1	296	33	4.87	1	1.77
	A0A8V0Y1U0	X-ray repair cross complementing 6 OS=Gallus gallus OX=9031 GN=XRCC6 PE=1 SV=1	2	1	1	1	649	74	6.15	1	1.69

ID 5983	Accession	Description	Coverage, %	# Peptides	# PSMs	# Unique Peptides	# AAs	MW, kDa	calc. pI	# Peptides *1	|Log Prob| *2
	A0A8V0Z0U1	phosphopyruvate hydratase OS=Gallus gallus OX=9031 GN=ENO1 PE=1 SV=1	8	3	5	3	445	48.7	7.06	3	17.38
	P53478	Actin, cytoplasmic type 5 OS=Gallus gallus OX=9031 PE=3 SV=1	10	4	4	4	376	41.8	5.48	4	16.35
	ALBU_BOVIN	(Common contaminant protein)	5	3	3	3	607	69.2	6.18	3	16.16
	Q5ZIQ3	Heterogeneous nuclear ribonucleoprotein K OS=Gallus gallus OX=9031 GN=HNRNPK PE=2 SV=1	8	2	2	2	427	47.2	6.47	2	14.2
	A0A8V1A5Y2	Heat shock 70kDa protein 8 OS=Gallus gallus OX=9031 GN=HSPA8 PE=1 SV=1	6	3	3	3	705	78	5.85	3	11.06
	P62801	Histone H4 OS=Gallus gallus OX=9031 GN=H4-VII PE=1 SV=2	21	2	4	2	103	11.4	11.36	2	10.37
	P09244	Tubulin beta-7 chain OS=Gallus gallus OX=9031 PE=2 SV=1	2	1	1	1	444	49.6	4.89	1	9.57
	A0A8V0YK12	ATP synthase subunit alpha OS=Gallus gallus OX=9031 GN=ATP5F1AW PE=1 SV=1	5	2	2	2	619	67.2	8.79	2	8.7
	A0A8V1AKG1	Ubiquitin B OS=Gallus gallus OX=9031 GN=LOC101747587 PE=4 SV=1	25	2	3	2	400	44.9	8.75	2	8.41
	Q5ZLN1	Phosphoglycerate mutase 1 OS=Gallus gallus OX=9031 GN=PGAM1 PE=1 SV=3	9	2	2	2	254	28.9	7.49	2	7.44
	P0CB50	Peroxiredoxin-1 OS=Gallus gallus OX=9031 GN=PRDX1 PE=1 SV=1	11	2	2	2	199	22.3	8.1	2	7.31
	P47826	Large ribosomal subunit protein uL10 OS=Gallus gallus OX=9031 GN=RPLP0 PE=2 SV=1	7	2	2	2	316	34.3	5.99	2	7.15
	Q5ZME1	Heterous nuclear ribonucleoprotein A2/B1 OS=Gallus gallus OX=9031 GN=HNRNPA2B1 PE=1 SV=1	4	1	1	1	349	37	8.65	1	6.14
	Q5ZMT0	14-3-3 protein epsilon OS=Gallus gallus OX=9031 GN=YWHAE PE=1 SV=1	5	1	1	1	255	29.2	4.74	1	6.12
	**A0A1D5PS82**	**Keratin OS=Gallus gallus OX=9031 GN=LOC121110032 PE=3 SV=1**	**12**	**1**	**2**	**1**	**98**	**10**	**6.24**	**1**	**6.01**
	A0A8V0XYP5	Heterogeneous nuclear ribonucleoprotein U OS=Gallus gallus OX=9031 GN=HNRNPU PE=1 SV=1	2	1	1	1	842	91.6	5.94	1	5.6
	Q5ZM98	Stress-70 protein, mitochondrial OS=Gallus gallus OX=9031 GN=HSPA9 PE=1 SV=1	2	1	1	1	675	73.1	6.43	1	5
	Q90835	Elongation factor 1-alpha 1 OS=Gallus gallus OX=9031 GN=EEF1A PE=2 SV=1	3	1	1	1	462	50.1	9.01	1	4.99
	A0A1D5NVI1	Large ribosomal subunit protein uL6 OS=Gallus gallus OX=9031 GN=RPL9 PE=1 SV=1	14	1	1	1	192	21.8	10.08	1	4.94
	A0A8V0ZG96	Pyruvate kinase OS=Gallus gallus OX=9031 GN=PKLR PE=1 SV=1	2	1	1	1	693	75.3	9.25	1	4.7
	A0A1L1RUW7	Deoxyuridine 5'-triphosphate nucleotidohydrolase OS=Gallus gallus OX=9031 GN=DUT PE=1 SV=2	6	1	1	1	187	20.2	8.88	1	4.53
	A0A3Q2U825	Small ribosomal subunit protein eS28 OS=Gallus gallus OX=9031 GN=RPS28 PE=1 SV=1	17	1	1	1	69	7.8	10.7	1	4.45
	A0A8V0ZPQ5	Karyopherin subunit beta 1 OS=Gallus gallus OX=9031 GN=KPNB1 PE=1 SV=1	2	1	1	1	885	98.3	4.81	1	4.38
	Q6ITC7	Small ribosomal subunit protein uS15 OS=Gallus gallus OX=9031 GN=RPS13 PE=2 SV=3	8	1	1	1	151	17.2	10.54	1	4.35
	Q5ZMJ6	ADP/ATP translocase OS=Gallus gallus OX=9031 GN=SLC25A4 PE=1 SV=1	3	1	1	1	298	32.9	9.73	1	4.34
	**A0A8V1A2T7**	**Keratin 18 OS=Gallus gallus OX=9031 GN=KRT18 PE=1 SV=1**	**2**	**1**	**1**	**1**	**429**	**46.9**	**5.19**	**1**	**4.22**
	Q8JG64	Protein disulfide-isomerase A3 OS=Gallus gallus OX=9031 GN=PDIA3 PE=2 SV=1	2	1	1	1	505	56.1	6.02	1	4.13
	P84175	Small ribosomal subunit protein eS12 OS=Gallus gallus OX=9031 GN=RPS12 PE=1 SV=2	8	1	1	1	132	14.5	7.21	1	4.07
	F1NN16	40S ribosomal protein S7 OS=Gallus gallus OX=9031 GN=RPS7 PE=1 SV=3	6	1	1	1	194	22.1	10.1	1	3.96
	A0A8V0X4F7	Fructose-bisphosphate aldolase OS=Gallus gallus OX=9031 GN=ALDOA PE=1 SV=1	6	1	1	1	192	20.7	8.16	1	3.93
	A0A8V0Z557	Heat shock protein HSP 90-alpha OS=Gallus gallus OX=9031 GN=HSP90AA1 PE=3 SV=1	2	1	1	1	734	84.5	5.08	1	3.92
	O13268	Proteasome subunit alpha type-7 OS=Gallus gallus OX=9031 GN=PSMA7 PE=2 SV=1	6	1	1	1	249	28.1	8.84	1	3.89
	A0A8V0ZA69	Serine/threonine-protein phosphatase PGAM5. mitochondrial OS=Gallus gallus OX=9031 GN=PGAM5 PE=1 SV=1	3	1	1	1	341	37.2	9.63	1	3.68
	A0A8V0Y5D3	Nucleophosmin OS=Gallus gallus OX=9031 GN=NPM1 PE=1 SV=1	7	1	1	1	307	34.1	4.83	1	3.68
	A0A8V1A3E8	H1.10 linker histone OS=Gallus gallus OX=9031 GN=LOC121111746 PE=4 SV=1	3	1	1	1	402	41.9	10.62	1	3.65
	P35062	Histone H2A-III OS=Gallus gallus OX=9031 PE=1 SV=2	15	1	1	1	129	14	10.9	1	3.63
	Q90593	Endoplasmic reticulum chaperone BiP OS=Gallus gallus OX=9031 GN=HSPA5 PE=1 SV=1	3	1	1	1	652	72	5.22	1	3.52
	A0A8V0XVK8	K Homology domain-containing protein OS=Gallus gallus OX=9031 GN=LOC420965 PE=1 SV=1	3	1	1	1	448	46.6	9.16	1	3.45
	A0A8V1AE41	Tyrosine 3-monooxygenase/tryptophan 5-monooxygenase activation protein gamma OS=Gallus gallus OX=9031 GN=YWHAG PE=1 SV=1	9	1	1	1	276	31.5	5.17	1	3.41
	A0A8V0Y1U0	X-ray repair cross complementing 6 OS=Gallus gallus OX=9031 GN=XRCC6 PE=1 SV=1	2	1	1	1	649	74	6.15	1	3.36
	P22451	Large ribosomal subunit protein uL18 OS=Gallus gallus OX=9031 GN=RPL5 PE=2 SV=2	5	1	1	1	297	34.1	9.72	1	3.36
	A0A8V0X7H3	Small ribosomal subunit protein RACK1 OS=Gallus gallus OX=9031 GN=RACK1 PE=3 SV=1	3	1	1	1	357	39.3	7.65	1	3.33
	A0A8V0Z4Q7	Large ribosomal subunit protein eL14 OS=Gallus gallus OX=9031 GN=RPL14 PE=1 SV=1	8	1	1	1	155	18.3	10.46	1	3.33
	**Q6PVZ1**	**Keratin. type I cytoskeletal 14 OS=Gallus gallus OX=9031 GN=KRT14 PE=2 SV=1**	**2**	**1**	**1**	**1**	**467**	**51**	**5.07**	**1**	**3.15**

ID 6220	Accession	Description	Coverage, %	# Peptides	# PSMs	# Unique Peptides	# AAs	MW, kDa	calc. pI	# Peptides *1	|Log Prob| *2
	**A0A1D5P6V7**	**Keratin OS=Gallus gallus OX=9031 GN=LOC121113098 PE=3 SV=1**	**16**	**5**	**8**	**5**	**98**	**9.9**	**7.52**	**5**	**19.35**
	Q5ZIQ3	Heterogeneous nuclear ribonucleoprotein K OS=Gallus gallus OX=9031 GN=HNRNPK PE=2 SV=1	15	4	4	4	427	47.2	6.47	4	13.44
	**A0A8V1AH20**	**Keratin OS=Gallus gallus OX=9031 GN=LOC100859427 PE=3 SV=1**	**14**	**3**	**4**	**3**	**98**	**9.9**	**7.94**	**3**	**12.77**
	A0A8V1A3F1	Tubulin alpha chain OS=Gallus gallus OX=9031 GN=LOC100859737 PE=3 SV=1	8	2	2	2	435	48.5	5.49	2	11.77
	ALBU_BOVIN	(Common contaminant protein)	8	3	3	3	607	69.2	6.18	3	11.04
	A0A8V0Z0U1	phosphopyruvate hydratase OS=Gallus gallus OX=9031 GN=ENO1 PE=1 SV=1	9	3	3	3	445	48.7	7.06	3	10.97
	P53478	Actin, cytoplasmic type 5 OS=Gallus gallus OX=9031 PE=3 SV=1	7	3	3	3	376	41.8	5.48	3	9.7
	A0A8V1AKG1	Ubiquitin B OS=Gallus gallus OX=9031 GN=LOC101747587 PE=4 SV=1	25	3	3	3	400	44.9	8.75	3	9.53
	P62801	Histone H4 OS=Gallus gallus OX=9031 GN=H4-VII PE=1 SV=2	21	2	3	2	103	11.4	11.36	2	9.29
	A0A8V0Z557	Heat shock protein HSP 90-alpha OS=Gallus gallus OX=9031 GN=HSP90AA1 PE=3 SV=1	3	2	2	2	734	84.5	5.08	2	5.11
	Q90835	Elongation factor 1-alpha 1 OS=Gallus gallus OX=9031 GN=EEF1A PE=2 SV=1	4	1	1	1	462	50.1	9.01	1	5.09
	A0A1D5NVI1	Large ribosomal subunit protein uL6 OS=Gallus gallus OX=9031 GN=RPL9 PE=1 SV=1	14	1	1	1	192	21.8	10.08	1	5.04
	A0A1D5PSE5	ATP-citrate synthase OS=Gallus gallus OX=9031 GN=ACLY PE=1 SV=1	2	1	1	1	1101	121.1	7.72	1	4.69
	Q5ZLN1	Phosphoglycerate mutase 1 OS=Gallus gallus OX=9031 GN=PGAM1 PE=1 SV=3	4	1	1	1	254	28.9	7.49	1	4.65
	A0A1L1RUW7	Deoxyuridine 5'-triphosphate nucleotidohydrolase OS=Gallus gallus OX=9031 GN=DUT PE=1 SV=2	6	1	1	1	187	20.2	8.88	1	4.4
	Q5ZMJ6	ADP/ATP translocase OS=Gallus gallus OX=9031 GN=SLC25A4 PE=1 SV=1	3	1	1	1	298	32.9	9.73	1	4.4
	A0A8V0XYP5	Heterogeneous nuclear ribonucleoprotein U OS=Gallus gallus OX=9031 GN=HNRNPU PE=1 SV=1	2	1	1	1	842	91.6	5.94	1	4.35
	A0A8V0YK12	ATP synthase subunit alpha OS=Gallus gallus OX=9031 GN=ATP5F1AW PE=1 SV=1	2	1	1	1	619	67.2	8.79	1	4.17
	A0A8V0Z4Q7	Large ribosomal subunit protein eL14 OS=Gallus gallus OX=9031 GN=RPL14 PE=1 SV=1	8	1	1	1	155	18.3	10.46	1	4.17
	**Q6PVZ1**	**Keratin. type I cytoskeletal 14 OS=Gallus gallus OX=9031 GN=KRT14 PE=2 SV=1**	**2**	**1**	**1**	**1**	**467**	**51**	**5.07**	**1**	**3.91**
	A0A8V0X7H3	Small ribosomal subunit protein RACK1 OS=Gallus gallus OX=9031 GN=RACK1 PE=3 SV=1	3	1	1	1	357	39.3	7.65	1	3.79
	A0A8V1A3E8	H1.10 linker histone OS=Gallus gallus OX=9031 GN=LOC121111746 PE=4 SV=1	3	1	1	1	402	41.9	10.62	1	3.78
	A0A8V0Y1U0	X-ray repair cross complementing 6 OS=Gallus gallus OX=9031 GN=XRCC6 PE=1 SV=1	2	1	1	1	649	74	6.15	1	3.73
	Q5ZM98	Stress-70 protein. mitochondrial OS=Gallus gallus OX=9031 GN=HSPA9 PE=1 SV=1	2	1	1	1	675	73.1	6.43	1	3.7
	A0A8V0YH91	Histone H2A OS=Gallus gallus OX=9031 PE=3 SV=1	21	1	1	1	137	14.8	10.74	1	3.54
	Q5ZMT0	14-3-3 protein epsilon OS=Gallus gallus OX=9031 GN=YWHAE PE=1 SV=1	4	1	1	1	255	29.2	4.74	1	3.46
	A0A8V1ACX6	T-complex protein 1 subunit gamma OS=Gallus gallus OX=9031 GN=CCT3 PE=1 SV=1	2	1	1	1	576	64	7.81	1	3.39
	A0A8V1A8R2	40S ribosomal protein S8 OS=Gallus gallus OX=9031 GN=RPS8 PE=1 SV=1	6	1	1	1	267	30.7	10.42	1	3.17
	A0A8V0YV66	Ribosomal protein S4. Y-linked 1 OS=Gallus gallus OX=9031 GN=RPS4Y1 PE=3 SV=1	4	1	1	1	342	37.3	10.42	1	3.16
	A0A8V0ZG96	Pyruvate kinase OS=Gallus gallus OX=9031 GN=PKLR PE=1 SV=1	2	1	1	1	693	75.3	9.25	1	3.16
	A0A8V0ZA69	Serine/threonine-protein phosphatase PGAM5. mitochondrial OS=Gallus gallus OX=9031 GN=PGAM5 PE=1 SV=1	3	1	1	1	341	37.2	9.63	1	3.03
	P0CB50	Peroxiredoxin-1 OS=Gallus gallus OX=9031 GN=PRDX1 PE=1 SV=1	5	1	1	1	199	22.3	8.1	1	2.99
	A0A1D5NVR2	Growth hormone receptor OS=Gallus gallus OX=9031 GN=GHR PE=3 SV=1	2	1	2	1	646	72.9	4.69	1	2.97
	A0A8V1A5Y2	Heat shock 70kDa protein 8 OS=Gallus gallus OX=9031 GN=HSPA8 PE=1 SV=1	2	1	1	1	705	78	5.85	1	2.8
	P26584	High mobility group protein B2 OS=Gallus gallus OX=9031 GN=HMGB2 PE=2 SV=2	6	1	1	1	207	23.8	8.38	1	2.79
	P50890	Small ribosomal subunit protein uS2 OS=Gallus gallus OX=9031 GN=RPSA PE=3 SV=1	6	1	1	1	296	33	4.87	1	2.77
	**A0A1L1RZQ0**	**Keratin OS=Gallus gallus OX=9031 GN=LOC121110033 PE=3 SV=2**	**10**	**1**	**1**	**1**	**98**	**9.9**	**7.52**	**1**	**2.47**
	Q5ZME1	Heterous nuclear ribonucleoprotein A2/B1 OS=Gallus gallus OX=9031 GN=HNRNPA2B1 PE=1 SV=1	3	1	1	1	349	37	8.65	1	2.33
	Q5F3W3	Mitogen-activated protein kinase 6 OS=Gallus gallus OX=9031 GN=MAPK6 PE=2 SV=1	2	1	1	1	721	82.7	5.07	1	2.11

Table S4: Spots from 2D SDS-PAGE with separated keratin analyzed by MS. Keratin related proteins were labelled bold. Contaminants were written in grey.
*1 by Search Engine: A18 PMI-Byonic. *2 by Search Engine PMI-Byonic A18

S5

Results of semi-quantitative analyses of keratins mass spectrometrical analyses of keratin extracted from 2D gel electrophoresis gel

Protein Rank	Description	|Log Prob|	Best |Log Prob|	Best score	Total Intensity	# of spectra	# of unique peptides	# of mod peptides	Cover age %	# AA's in protein	Protein DB number	Chart name	iBAQ score	Percentage proportion, %
1	>sp|P20307|KRFD_CHICK Feather keratin 3 OS=Gallus gallus OX=9031 PE=3 SV=2	264,70	5,50	605,10	1453055153,9	426	133	18	95,92	98	1926	Feather keratin 3	10925226,7	**36,22936066**
2	>sp|O13152|KRFJ_CHICK Beta-keratin-related protein OS=Gallus gallus OX=9031 GN=BKJ PE=3 SV=3	125,71	5,25	490,00	234187529,2	120	54	0	65,14	109	1898	Beta-keratin	4336806,1	**14,38136857**
3	>sp|P20308|KRFA_CHICK Feather keratin 4 OS=Gallus gallus OX=9031 PE=3 SV=2	38,14	4,57	422,40	81539726,6	41	18	0	38,78	98	1927	Feather keratin 4	4529984,81	**15,02197233**
4	>sp|P04458|KRFB_CHICK Feather keratin 2 OS=Gallus gallus OX=9031 PE=3 SV=3	12,65	2,33	427,30	114254012,5	38	35	35	56,12	98	1911	Feather keratin 2	3264400,36	**10,82514266**
12	>sp|P02450|KRFC_CHICK Feather keratin 1 OS=Gallus gallus OX=9031 PE=3 SV=2	5,11	2,09	270,70	4480806,5	5	4	2	36,73	98	1908	Feather keratin 1	1120201,63	**3,71472279**
	All other hits - defined or undefined				1207779484		202					Contaminant	5979106,36	**19,82743298**
												Total	30155726	

Table S5: Semi-quantitative analyses of keratin proportions based on iBAQ scores.

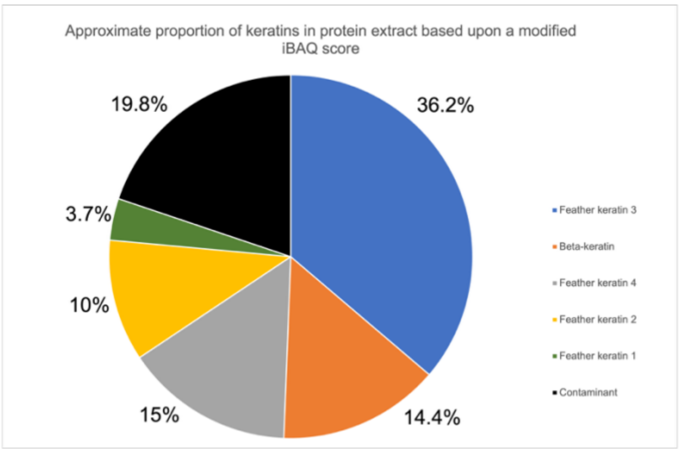

Fig. S5: Results of the keratin proportions. With about 36% of the keratin particles, feather keratin 3 was determined to be the main fraction, whereby feather keratin 1 has the smallest ratio with about 3.7%.

However, the modified process used the number of unique and observable Proteinase K peptides. This might have unintended quantification issues in larger, more complex samples due to the significantly more complex peptide pools generated by such an aggressive and relatively untargeted protease such as Proteinase K. But, taking into account that with the conserved nature of the sizes of the observed protein extracts (4 of the 5 have 98 amino acids, with the other protein having 109 AAs), in this case, we assume that the untargeted nature of the protease imported no measurable bias into the resultant proportions. In other words, what affects one, will affect them all equally. However, it should be stressed that 'spectral counting' methods such as this were not as accurate as fully quantitative methods such as isotopic labelling processes or multiple reaction monitoring (MRM) methods and should be treated as such.

S6

Results of particle size (DLS) and zeta potential determination including pH titration

The size of the colloidal keratin particles out of *n* = 10 batches (five batches from two slaughterhouses) was analyzed by dynamic light scattering (DLS) and each measurements was carried out as triplicates with the following results:

PDI	Intensity Peak 1 [9]	Intensity Peak 2 [9]	Intensity Peak 1 Area [%]	Intensity Peak 2 Area [%]	Z-pot. (mV) NaCl	Z-pot. (mV) DI water
0.414 ± 0.045	299 ± 52	47 ± 10	92 ± 5	8 ± 5	-25.0 ± 2.6	-40.4 ± 3.9

Table S6: Data of DLS measurements (173° measuring angle) and zeta potential for the colloidal keratin solutions of *n* = 10 batches. Each batch was measured as independent triplicate (*n* = 3) with both methods. 0.1 mM colloidal keratin solutions (dilute in DI water) were used for the DLS and 0.3 mM (dilute in 10 mM NaCl) or 0.1 mM (dilute in DI water) protein suspensions for the determination of zeta potential. (PDI – Polydispersity index, Intensity Peak [9] – Peak mean by intensity ordered by area [9], Intensity Peak Area [%] – Peak area by intensity ordered by area [%], Z-pot. – Zeta potential).

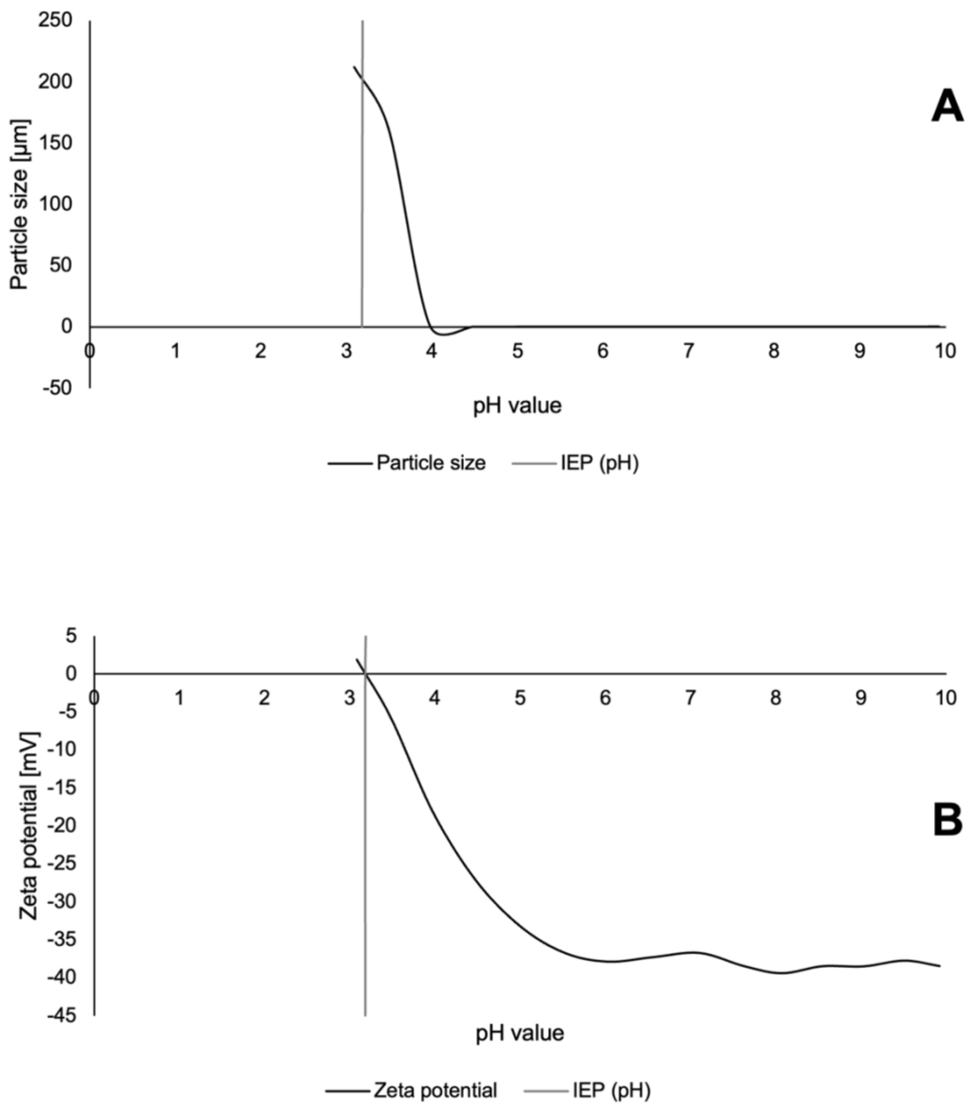

Fig. S6: A: Particle size of keratin from pH 3 to 10. The size of the keratin particles per pH value were ascertained by titration of a 0.1 mM keratin solution (particles diluted in DI water) (n = 3). B: Zeta potential of keratin from pH 3 to 10. The zeta potential of the keratin particles per pH value were ascertained by titration of a 0.1 mM keratin solution (particles diluted in DI water) (n = 3).

S7

<u>Intention to evaluate the molecular weight of the keratin particles via SEC-MALLS</u>

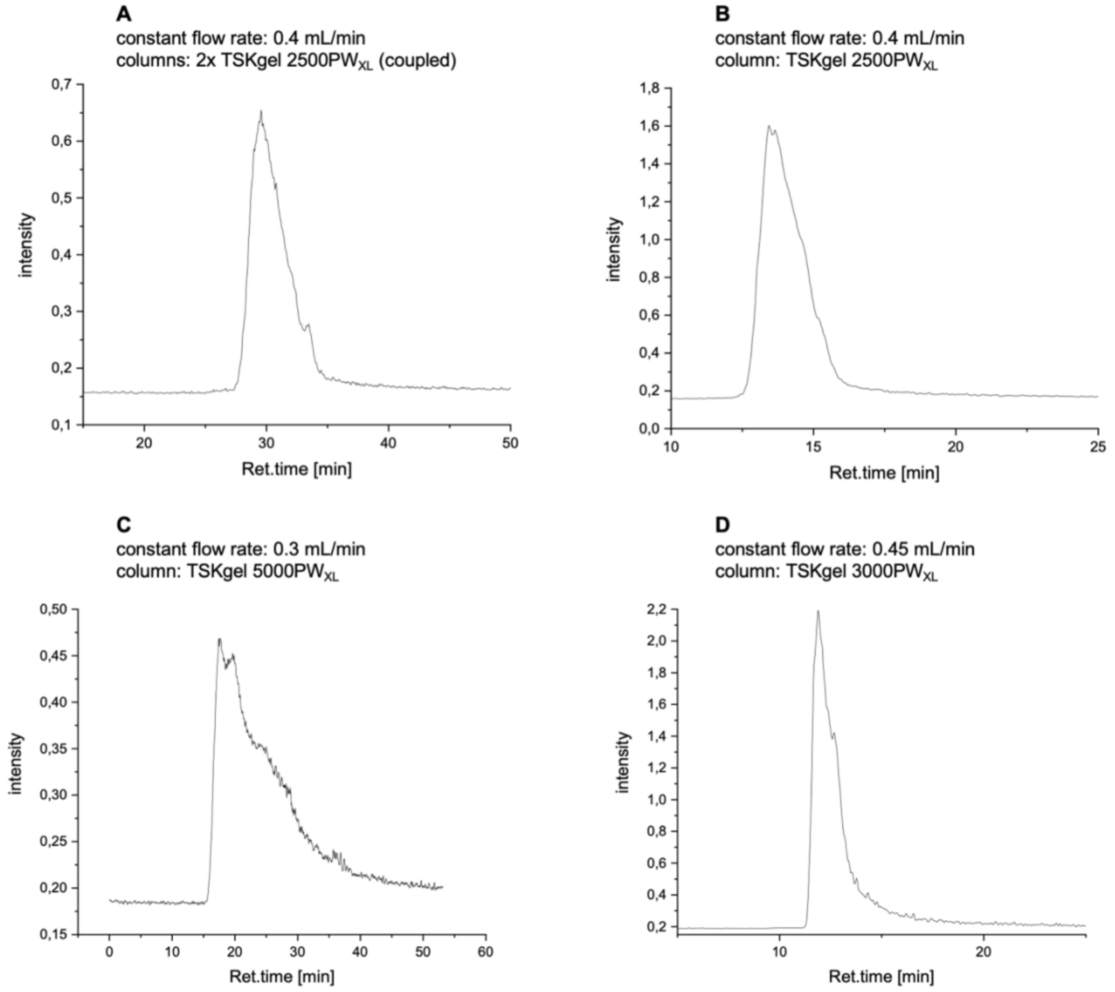

Figure S7: SEC-MALLS chromatogram (angle: 90 °) of keratin (1.0 mg/mL), mobile phase: ultrapure water (containing 0.05% sodium azide) with different columns and flow rates (A-D), with *n* = 3 per measurement setting.

S8

Results of the HET-CAM tests of keratin powder and colloidal dispersion

Table S8: HET-CAM data of test compounds consisting of the pure powder, a 2.88 mg/ml colloidal keratin solution in DI water, 1% SDS solution as positive control, and *Aqua ad injectionem* as negative control. (C = Coagulation, H = Hemorrhage, IS = Irritation score, L = Vascular lysis, SG = degree of severity). All test solutions, keratin dispersion and keratin powder were tested independently (*n* = 6).

**Test compounds**	**IS-H**	**IS-L**	**IS-C**	**IS-G**	**SG-H**	**SG-L**	**SG-C**	**Irritant potential**
1% SDS solution	2.89±0.15	0.90±0.06	6.01±0.59	9.80±0.65	1.67±0.43	0.42±0.02	3.00±0.09	strong
Aqua ad injectionem	0.00	0.00	0.00	0.00	0.00	0.00	0.00	no
2.88 mg/ml keratins in DI water	0.00	0.00	0.00	0.00	0.00	0.00	0.00	no
10 mg keratin particles (powder)	0.00	0.00	0.00	0.00	0.00	0.00	0.00	no

S9

Results of the microbiological stability of the keratin powder

Determination		Test method	Result	Evaluation
TAMC	Microbiological testing of non-sterile products	Ph. Eur. 6.0 (6.0/2.06.12.00)	1,7 x 10^2 CFU/g	Target value: < 10^3 CFU/g<br>Target value achieved
TYMC	Microbiological testing of non-sterile products	Ph. Eur. 6.0 (6.0/2.06.12.00)	10 CFU/g	Target value: < 10^2 CFU/g<br>Target value achieved

Table S9: Microbiological stability of keratin powder following test method Ph. Eur. 6.0 (6.0/2.06.12.00). (TAMC – Total Aerobic Microbial Count, TYMC – Total Yeast and Mold Count). Results were presented in colony forming units, CFU. Each test was performed twice (*n* = 2).

References

[[1] Shuihong Zhu, *et al.*, *Biopolymeric Ionotronics Based on Biodegradable Wool Keratin*. Advanced Materials, 2025. **37**(22): p. 2414191. https://doi.org/10.1002/adma.202414191

[2] Xiang Mi, *et al.*, *Transferring feather wastes to ductile keratin filaments towards a sustainable poultry industry*. Waste Management, 2020. **115**: p. 65–73. https://doi.org/10.1016/j.wasman.2020.07.022

[3] Xiang Mi, Helan Xu, and Yiqi Yang, *Submicron amino acid particles reinforced 100% keratin biomedical films with enhanced wet properties via interfacial strengthening*. Colloids and Surfaces B: Biointerfaces, 2019. **177**: p. 33–40. https://doi.org/10.1016/j.colsurfb.2019.01.043

[4] Helan Xu, Zhuanzhuan Ma, and Yiqi Yang, *Dissolution and regeneration of wool via controlled disintegration and disentanglement of highly crosslinked keratin*. Journal of Materials Science, 2014. **49**(21): p. 7513–7521

[5] A. Grazziotin, F. A. Pimentel, E. V. De Jong, and A. Brandelli, *Nutritional improvement of feather protein by treatment with microbial keratinase*. Animal Feed Science and Technology, 2006. **126**(1): p. 135–144. https://doi.org/10.1016/j.anifeedsci.2005.06.002

[6] B. Ma, X. Qiao, X. Hou, and Y. Yang, *Pure keratin membrane and fibers from chicken feather*. Int J Biol Macromol, 2016. **89**: p. 614–21. 10.1016/j.ijbiomac.2016.04.039

[7] Helan Xu and Yiqi Yang, *Controlled De-Cross-Linking and Disentanglement of Feather Keratin for Fiber Preparation via a Novel Process*. ACS Sustainable Chemistry & Engineering, 2014. **2**(6): p. 1404–1410. 10.1021/sc400461d

[8] Shuang Xia*, et al.*, *Using a Functional Wool Keratin Photoresist to Build Iridescent and Fluorescent 3D Micro-Pattern for Dual-Mode Optical Anti-Counterfeiting*. Small, 2025. **21**(25): p. 2502166. https://doi.org/10.1002/smll.202502166

[9] National Institute of Environmental Health Sciences, *ICCVAM Test Method Evaluation Report - in vitro cytotoxicity test methods for estimating starting doses for acute oral systemic toxicity testing*. 2006.
